# Developing a medical device-grade T_2_ phantom optimized for myocardial T_2_ mapping by cardiovascular magnetic resonance

**DOI:** 10.1186/s12968-023-00926-z

**Published:** 2023-03-20

**Authors:** Constantin-Cristian Topriceanu, Massimiliano Fornasiero, Han Seo, Matthew Webber, Kathryn E. Keenan, Karl F. Stupic, Rüdiger Bruehl, Bernd Ittermann, Kirsty Price, Louise McGrath, Wenjie Pang, Alun D. Hughes, Reza Nezafat, Peter Kellman, Iain Pierce, James C. Moon, Gabriella Captur

**Affiliations:** 1 Barts Heart Center, Barts Health NHS Trust, West Smithfield, London, ECIA 7BE UK; 2 Institute of Cardiovascular Science, University College London, Huntley Street, London, WC1E 6DD UK; 3 UCL Medical School, 74 Huntley Street, London, WC1E 6DE UK; 4 Department of Cardiology, Center for Inherited Heart Muscle Conditions, Royal Free London NHS Foundation Trust, Pond Street, London, NW3 2QG UK; 5 Medical Research Council Unit for Lifelong Health and Ageing at UCL, 1-19 Torrington Place, London, WC1E 7HB UK; 6 National Institute of Standards and Technology (NIST), 325 Broadway, Boulder, CO 80305 USA; 7 Physikalisch-Technische Bundesanstalt (PTB), Abbestraße 2-12, 10587 Berlin, Germany; 8 UCL Bloomsbury Center for Clinical Phenotyping, London, WC1E 6HX UK; 9 Resonance Health (RH), 141 Burswood Road, Burswood, WA 6100 Australia; 10 Department of Medicine (Cardiovascular Division), Beth Israel Deaconess Medical Center, Harvard Medical School, 330 Brookline Ave, Boston, MA 02215 USA; 11 National Heart, Lung and Blood Institute, National Institutes of Health (NIH), Rockville Pike, Bethesda, MD 20892 USA; 12 Institute of Cardiovascular Science, Consultant Cardiologist in Inherited Heart Muscle Conditions, University College London, Gower Street, London, WC1E 6BT UK

**Keywords:** T_1_ mapping, T_2_ mapping, Phantom, Quality control

## Abstract

**Introduction:**

A long T_2_ relaxation time can reflect oedema, and myocardial inflammation when combined with increased plasma troponin levels. Cardiovascular magnetic resonance (CMR) T_2_ mapping therefore has potential to provide a key diagnostic and prognostic biomarkers. However, T_2_ varies by scanner, software, and sequence, highlighting the need for standardization and for a quality assurance system for T_2_ mapping in CMR.

**Aim:**

To fabricate and assess a phantom dedicated to the quality assurance of T_2_ mapping in CMR.

**Method:**

A T_2_ mapping phantom was manufactured to contain 9 T_1_ and T_2_ (T_1_|T_2_) tubes to mimic clinically relevant native and post-contrast T_2_ in myocardium across the health to inflammation spectrum (i.e., 43–74 ms) and across both field strengths (1.5 and 3 T). We evaluated the phantom’s structural integrity, *B*_0_ and *B*_1_ uniformity using field maps, and temperature dependence. Baseline reference T_1_|T_2_ were measured using inversion recovery gradient echo and single-echo spin echo (SE) sequences respectively, both with long repetition times (10 s). Long-term reproducibility of T_1_|T_2_ was determined by repeated T_1_|T_2_ mapping of the phantom at baseline and at 12 months.

**Results:**

The phantom embodies 9 internal agarose-containing T_1_|T_2_ tubes doped with nickel di-chloride (NiCl_2_) as the paramagnetic relaxation modifier to cover the clinically relevant spectrum of myocardial T_2_. The tubes are surrounded by an agarose-gel matrix which is doped with NiCl_2_ and packed with high-density polyethylene (HDPE) beads. All tubes at both field strengths, showed measurement errors up to ≤ 7.2 ms [< 14.7%] for estimated T_2_ by balanced steady-state free precession T_2_ mapping compared to reference SE T_2_ with the exception of the post-contrast tube of ultra-low T_1_ where the deviance was up to 16 ms [40.0%]. At 12 months, the phantom remained free of air bubbles, susceptibility, and off-resonance artifacts. The inclusion of HDPE beads effectively flattened the *B*_0_ and *B*_1_ magnetic fields in the imaged slice. Independent temperature dependency experiments over the 13–38 °C range confirmed the greater stability of shorter vs longer T_1_|T_2_ tubes. Excellent long-term (12-month) reproducibility of measured T_1_|T_2_ was demonstrated across both field strengths (all coefficients of variation < 1.38%).

**Conclusion:**

The T_2_ mapping phantom demonstrates excellent structural integrity, *B*_0_ and *B*_1_ uniformity, and reproducibility of its internal tube T_1_|T_2_ out to 1 year. This device may now be mass-produced to support the quality assurance of T_2_ mapping in CMR.

**Supplementary Information:**

The online version contains supplementary material available at 10.1186/s12968-023-00926-z.

## Introduction

Cardiovascular magnetic resonance (CMR) allows noninvasive myocardial tissue characterization. Fully quantitative T_2_ mapping techniques expose not only regionality, but also diffuse changes in T_2_, offering the prospect of redefining disease (disease vs. normal) and monitoring interval change. However, the measured T_2_ values differ by parameters such as temperature, field strength, type of scanner and CMR sequence. The lack of protocol standardization has hampered the definition of myocardial T_2_ ranges in health, the pooling of multi-center mapping data into generalizable outputs, and the robust conduct of longitudinal studies that serially measure T_2_. The Society for Cardiovascular Magnetic Resonance (SCMR)’s current recommendation is to perform stratified statistical analysis to adjust for site scan characteristics [1]. However, as a first step towards better standardization, an internationally accepted reference object for CMR T_2_ mapping is desirable [2].

In clinical practice, myocardial T_2_ can vary with age [3, 4], sex [3–5], myocardial region (e.g., shorter T_2_ in apical segments) [5, 6], T_2_ mapping sequence (e.g., shorter T_2_ with T_2_-prepared balanced steady state free precession [bSSFP] compared to gradient and spin echo (SE)) [6] and field strength (lower at 3 T) [6]. In the literature, the normal values for native myocardial T_2_ in health have been provided as mean ± 1 standard deviation (SD) (though a 95% reference range is approximately ± 2 SD). Although there have been multiple attempts to establish age and sex corrected normal values for T_2_ [3, 7, 8], currently no widely accepted reference ranges exist. Similarly, the myocardial T_2_ values in inflammation are still debated, but they seem to lie in the ~ 55 to 74 ms interval at 1.5 T [2].

The T_1_ Mapping and Extracellular Volume standardization (T1MES®) phantom [9] previously developed by our research group, provided a roadmap for developing a quality assurance medical device system for T_1_ mapping and this has now been extensively validated [10]. Yet T1MES® was primarily a T_1_ mapping phantom, designed to cover the range of blood and myocardial T_1_ before and after the administration of gadolinium-based contrast agents (GBCA). Its T_2_ coverage was therefore limited to just 6 T_2_ values from 42 to 243 ms but none in the ~ 55 to 74 ms interval [9] meaning it provided a poor coverage of long native myocardial T_2_ that we typically measure in the acutely inflamed myocardium [2]. The use of T1MES® as a T_2_ quality assurance device, is therefore hindered by the fact that it does not offer a granular enough representation of the relevant T_2_ values across the health to inflammation spectrum (i.e., ~ 43 ms–74 ms). The hypertrophic cardiomyopathy registry (HCMR) phantom [11] was also designed for T_1_ mapping and covers only two myocardial T_2_ values (57 ms and 75 ms). Lastly, although the International Society of Magnetic Resonance in Medicine (ISMRM)/National Institute of Standards and Technology Laboratory (NIST) phantom [12] provides wide T_2_ coverage with 14 vials spanning T_2_ from 5 to 940 ms, only two of these are relevant for human myocardium (45 ms and 65 ms). Therefore, the existing CMR phantoms fall short of capturing clinically valid myocardial T_2_ values meaning that the cardiovascular T_2_ mapping community does not have a robust quality assurance reference object.

Using the collaboration and expertise gained through the T1MES® programme, we sought to design a T_2_ mapping phantom that could be used interchangeably at both 1.5 T and 3 T, and that reflects clinically relevant native and post-GBCA T_2_ in myocardium across the spectrum of health and disease.

## Materials and methods

### Collaboration process

The design collaboration has been previously described in the literature [9]. Briefly, it consisted of clinicians, physicists, national metrology institutes (the NIST and the German Physikalisch-Technische Bundesanstalt [PTB]) and a medium enterprise (the Australian Resonance Health [RH]).

### Phantom and tube composition

Currently, there is no ideal material for phantom manufacture. As a first step, materials were filtered based on flow properties since fluid movement during imaging can introduce uncertainty in the T_2_^*^ to T_2_ conversion [13]. Given their viscosity, gels (e.g., agarose, gelatin, silicone, polyacrylamide, etc.) are preferable as they are not prone to fluid movement within an image slice during inversion recovery. Although the long-term stability of gels is limited because of gel contraction leading to gaps, we opted to use an agarose-based gel for phantom manufacture as this has been shown to be stable for 1–2 years [10, 14]. As microbial action can affect long-term stability, decontaminated high purity water was used. Although there are many available paramagnetic ions (e.g., copper, iron, manganese etc.), we chose nickel (Ni^2+^) given its lesser dependence on frequency and temperature [15, 16]. Thus, our phantom was a diamagnetic matrix consisting of an agarose-gel (polysaccharide agarose powder with low endosmotic flow for electrophoresis, molar ratio ≤ 0.07, Acros Organics) prepared with high purity deionized water (Ibis Technology) doped with paramagnetic nickel di-chloride (NiCl_2_).

Each phantom contained 9 tubes (#60.9922.212, 30 ml from Sarstedt, Numbrecht, Germany) filled with the gel matrix described above (i.e., the inner matrix fill). The tubes were tightly screw-capped to prevent leaks. Since the concentration of the gelling agent [17] and the paramagnetic ion concentration [15] are inversely correlated with T_2_ and T_1_ (T_1_|T_2_) respectively, a gel tube with any required T_1_|T_2_ can be theoretically designed. The T_1_ and T_2_ interdependence of agarose and nickel has been previously described [9]. Briefly, different concentrations of NiCl_2_, agarose and water were prepared, transferred into preheated nuclear magnetic resonance (NMR) tubes (to avoid instant setting), allowed to set, analyzed at 22 °C using a non-imaging 1.4 T Bruker (Billerica, Massachusetts, USA) Minispec mq60 (60 MHz) relaxometer, and T_1_|T_2_ were recorded following exponential fitting. Assuming a linear relationship between ingredients and relaxation rates (i.e., 1/T_1_ and 1/T_2_) [18], the ingredients required for any T_1_|T_2_ tube could thus be calculated. Thus, 9 unique stock solutions were constructed providing the clinically relevant native and post-GBCA T_1_|T_2_ tube combinations observed in myocardium across the spectrum of health and disease (Table 1). A detailed description of the linear models used for longitudinal and transverse relaxation rates in terms of the ingredients agarose and NiCl_2_, and of the exponential fitting has been previously published [9].

**Table 1:** Measured T_1_|T_2_ myocardial values for the 9 tubes and outer matrix fill

	T1 (ms)			T2 (ms)			Agarose (%)	Ni^2+^ (mM)
	1.4 T	1.5 T	3 T	1.4 T	1.5 T	3 T		
Short T_2_ native myo at both 1.5 T and 3 T (A)	821	803	807	40	35	34	1.261	3.139
Medium T_2_ native myo at 1.5 T (B)	978	982	988	50	43	41	0.969	2.511
Medium T_2_ native myo at 3 T (C)	1122	1073	1137	48	42	40	0.773	2.639
Long T_2_ native myo at 1.5 T (D)	1083	1090	1130	71	60	59	0.821	1.775
Long T_2_ native myo at 3 T (E)	1237	1225	1228	70	61	59	0.649	1.791
Long-normal T_2_ native myo at 1.5 T (F)	1030	1015	1019	60	52	50	0.882	2.081
Very long T_2_ native myo at 3 T (G)	1295	1287	1302	82	70	68	0.594	1.562
Mildly long T_1_ & T_2_ native myo at 1.5 & 3 T (H)	1221	1182	1217	60	52	51	0.664	2.139
Medium T_1_ & T_2_ post-GBCA* myo at 1.5 & 3 T (I)	440	435	445	47	41	40	2.840	2.502
Outer gel matrix fill	850	-	-	140	-	-	1.155	0.780

All the T_1_|T_2_ presented in this table were measured in our final phantom (*n* = 1). T_1_|T_2_ at 1.4 T were measured by a Bruker minispec mq60 relaxometer (22 °C) at Resonance Health laboratory in Australia; T_1_ and T_2_ at 1.5 T and 3 T were measured by inversion recovery gradient echo and single-echo spin echo at University College London at baseline. The definitions of ‘short’, ‘medium’, ‘long-normal’, ‘long’, ‘mildly long’ and ‘very long’ are subjective and relative to the average normal native myocardial T_2_ in health

^*^The post-contrast myocardial T_1_ behavior being modelled here is based on the published literature around the use of Dotarem (Gadoterate meglumine, Guerbet, France) so the effects may not be generalizable to other GBCAs

*GBCA* gadolinium-based contrast agent;* myo* myocardium; ms, milliseconds

These 9 tubes were contained within a plastic bottle and the inter-tube space packed with the outer matrix fill which consisted of a similar NiCl_2_ doped agarose-gel matrix as described above, but additionally containing high-density polyethylene (HDPE) beads. Regarding the choice of the outer matrix fill T_1_|T_2_ properties_,_ we selected the combination that yielded the lowest bSSFP stabilization artefacts at both 1.5 T and 3 T based on *B*_1_ uniformity experiments described below. We chose HDPE beads as compared to sodium chloride or other plastic microbeads as they were better at flattening the *B*_1_ field (experiments previously reported [9]). Since HDPE beads have a similar diamagnetism to the gel, the beads do not impact the *B*_0_ field.

### Structural integrity

Gel integrity and aging were checked at baseline (i.e., on receipt of the phantom in the UK post manufacture in Australia) and at 12-months. This was through the manual inspection of localizers and a high-resolution, isotropic, three-dimensional (3D) gradient echo sequence (0.42mm^3^) acquired on two Siemens CMR systems (Siemens Healthineers, Erlangen, Germany) at the University College London (UCL) Bloomsbury Center for Clinical Phenotyping using Magnetom Aera 1.5 T operating VE11C-SP01 and Magnetom Prisma 3 T operating VE11C-SP01, both with 18-channel phased-array chest coils. The latter sequence acquired two overlapping slabs (due to scanner software constraints), each with two directions of phase encoding. It also had a long repetition time (TR = 17 ms) and narrow pixel bandwidth (250 Hz/pixel) for improved signal-to-noise ratio (SNR). This sequence had weak T_1_ and T_2_ image contrast and was only used for structural examination.

### ***B***_0_ and*** B***_1_ uniformity

The phantom is composed of both paramagnetic constituents (such as Ni^2+^ that are attracted to *B*_0_ because they have at least one unpaired electron) and diamagnetic constituents (such as agarose that are repelled by *B*_0_ as all their electrons are paired). Since the concentration of Ni^2+^ is small, the paramagnetic effect of the phantom is < 10%. Thus, the *B*_0_ distortion caused by phantom components arises mainly from electronic diamagnetism. Although increasing the paramagnetic ion concentration would have counterbalanced the diamagnetism, this would have resulted in an excessive shortening of the relaxation times.

**Fig. 1:**
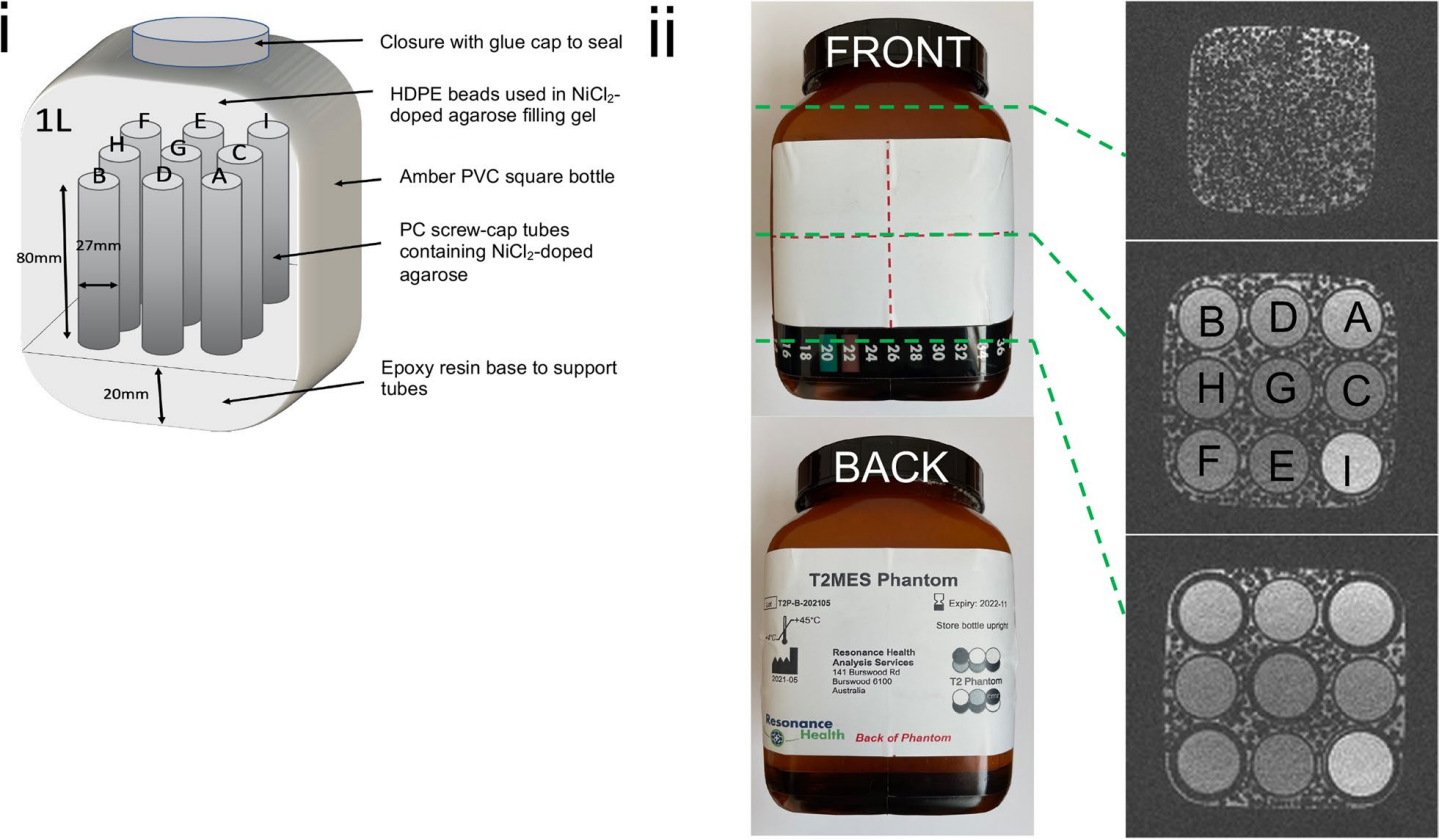
**i** Schematic (not to scale) showing the internal and external phantom structure. **ii** Phantom front view showing isocenter line and liquid crystal display thermometer. *HDPE* = *high-density polyethylene; PVC* = *polyvinyl chloride; PC* = *polycarbonate*

Regarding phantom design, the ideal shape would have been Lorentz uniform (e.g., ellipsoid body) to avoid susceptibility-induced magnetostatic field perturbations, but such perfectly ellipsoidal geometry is difficult to mass produce. Although many phantoms are cylindrical, there are off-resonance artefacts even if the phantom is co-axially aligned with B_0_ [19]. To compromise from a geometric point of view, our phantom’s outer body shape along the z-axis was fairly ellipsoidal (Fig. 1i) but square in cross-section (Fig. 1ii) as it consisted of a rounded-edged, short, hollow, wide necked and leakproof brown-transparent poly vinyl chloride (PVC) bottle with a melting temperature of 140 °C. The volume of the phantom was 1 L, its length was 13 cm and inner body cross section was 10 cm by 10 cm. As the bottle base, cap and edges were prone to off-resonance errors, internal tubes were located near to the center of the bottle and fixed on top of a 20 mm layer of Epoxycast clear casting resin (Barnes, NSW, Australia). The choice of a resin layer height of 20 mm was guided by T_2_ mapping bSSFP experiments which showed an off-resonance band compromising the lower 15 mm of the phantom body. To minimize field inhomogeneities at the bottle edges, tubes with long T_1_|T_2_ were arranged more centrally and avoided the corners. The physical length of the 9 T_1_|T_2_ tubes was the same. *B*_0_ field uniformity as a measure of off-resonance at both 1.5 T and 3 T was mapped using a single-echo gradient echo sequence, based on the phase difference between known echo times (TE) [20].

In the radiofrequency (RF) *B*_1_ field, the water dipole moment rotates leading to displacement current. Using Maxwell’s equations, the rate of the displaced current to conducted current is $Q= \frac{\mathrm{\omega \varepsilon }}{\upsigma }$ (where ω is the angular frequency, ε is the permittivity and σ the conductivity). Thus, *B*_1_ uniformity across the phantom body, could have been improved by either decreasing the permittivity or increasing the conductivity of the outer matrix fill. As per our previous work [9], we opted to decrease the permittivity and did so by densely packing oblate spheroidal HDPE beads (3 mm polar axis by 4.2 mm equatorial diameter) into the outer matrix fill. Each bead consisted of smooth, semi-translucent, colorless HDPE with a melting index > 60 °C (HDPE Marlex HHM 5502 BN, Chevron Phillips Chemical Company LP, Woodlands, Texas, USA). *B*_1_ homogeneity was evaluated through flip angle (FA) maps derived by the double angle method (i.e., 60° and 120°) using short (i.e., 4 ms) sinc (− 3π to + 3π) slice excitation width via long TR (i.e., 8 s) scanning.

### Reference T_1_ and T_2_

CMR studies on the T_2_ phantom at baseline were performed using the 1.5 T Aera and 3 T Prisma (Siemens Healthineers) scanners at UCL. The scan protocol was identical for the two field strengths and consisted of inversion recovery (IR) gradient echo (GRE) for measuring reference T_1_ (11 inversion times [ms]: 20, 50, 100, 200, 400, 600, 800, 1000, 1300, 1700, 2100; FA: 90°; TR: 10 s; resolution: 1.8 mm × 11.8 mm; slice thickness: 8 mm) and single-echo SE for measuring reference T_2_ (10 TE [ms]: 10, 20, 30, 40, 50, 60, 80, 100, 125, 150; FA: 90°; TR: 10 s; resolution: 1.8 mm × 11.8 mm; slice thickness: 8 mm), both at 22 oC.

### Temperature dependence of T_1_ and T_2_

Temperature dependency experiments on T_1_|T_2_ values were carried out at:

- *NIST* at 5 temperatures between 20.0 °C and 36.6 °C using an Agilent 3 T small bore scanner. T_1_ was measured by IRSE (TR: 10 s; TI [ms]: 50, 75, 100, 125, 150, 250, 500, 1000, 1500, 2000, 3000, 6000) and T_2_ by SE (TR: 10 s; TE [ms]: 14, 28, 56, 112);
- *PTB* at 6 temperatures between 13.5 °C and 38.8 °C using a 3 T Magnetom Verio system (VB17; Siemens Healthineers) and a 12-channel head coil. T_1_ was measured by inversion recovery spin echo (IRSE, TR: 8 s; TI: 25–4800 ms) and T_2_ by SE (TR:3 s; TE: 24–400 ms).

At both centers, the scan resolution was 0.5 mm x 0.5 mm^2^ and slice thickness was 2 mm. In addition, temperatures were measured using fiber optic probes.

### Reproducibility

Short-term reproducibility experiments were performed using 3 repeats of T_1_ mapping and T_2_ mapping sequences on two final phantom prototypes (#Ci and #Cii) manufactured 12 months apart using independent stock solutions.

Long-term reproducibility experiments were performed to assess the variability of T_1_ and T_2_ measurements at baseline and at 12 months in one phantom (#Ci).

All tests were performed on the UCL 1.5 T and 3 T scanners both operating VE11A. T_1_ mapping was by a 5 s(3 s)3 s 256-matrix RR = 900 ms variant of modified Look-Locker inversion recovery (MOLLI) adapted for native T_1_ mapping (1.5 T FA: 35°; 3 T FA: 20°; both Siemens WIP 1041B, acquired resolution: 1.4 mm × 1.9 mm and slice thickness: 8 mm). T_2_ mapping was by a T_2_-prepared T_2_ mapping sequence (bSSFP) at 1.5 T and 3 T (both FA: 70°; acquired resolution: 1.9 mm × 2.3 mm; slice thickness 8 mm). Each tube’s T_1_|T_2_ were calculated in the reconstructed T_1_|T_2_ pixel-wise maps as the mean signal intensity values obtained from fixed diameter circular regions of interest (ROI) automatically placed in the central 50% radius of each tube. However, manual corrections were applied if appropriate to ensure optimal ROI centering in each tube.

Each scan session replicated the prescribed phantom set-up, with simulated electrocardiogram (ECG) at 67 beats per minute (ECG R-wave to R-wave interval: 900 ms). Details regarding the phantom position and adjustments before scanning, and phantom storage instructions can be found in the user manual (Additional file 1).

### Statistical analysis

Statistical analysis was performed in R (version 4.0, R Foundation for Statistical Computing, Vienna, Austria). Curve fitting and ROI measurement was performed in MATLAB (R2020a, Mathworks, Natick, Massachusetts, USA). Distribution of data were assessed on histograms and normality checks were performed using the Shapiro–Wilk test. Continuous variables are expressed as mean ± 1 SD. Details for how we defined the model that describes the relation between ingredients and relaxation rates by fitting a surface for T_1_ and T_2_, and using the linear least-squares approach, are provided in our previously published phantom work [9]. The reproducibility between repeated scans was assessed through the coefficients of variation (CoV).

## Results

### Prototype testing and final phantom design

Three sequential prototypes (#A, #B, #C) were initially tested for *B*_0_ and *B*_1_ field inhomogeneities. Briefly, during the first two iterations (#A, #B), we reduced the bottle volume compared to T1MES® to reduce the artefacts and experimented with the use of 7 instead of 9 internal tubes arranged in a circular array (image not shown). However, this configuration was not stable and tube position shifts were observed after shipping, so the third and final prototype (#Ci) was constructed with a 3 × 3 array of 9 internal tubes, of slightly smaller diameter (24 mm) compared to T1MES®. Each center received a #Ci phantom, and provided quantitative and visual data for this manuscript. To study the reliability of manufacture and verify short-term reproducibility, another final prototype (#Cii) was manufactured 12 months after the manufacture of #Ci and sent to UCL to test the reliability of the production process having redone the 9 independent stock solutions.

A schematic representation of the phantom showing its internal and external structure is displayed in Fig. 1i. The actual phantom front and back views displaying cross-sectional slices of the isoelectric line, bottle cap and bottle base (above the epoxy resin base) are shown in Fig. 1ii. Tube arrangement is such that the more temperature-dependent and therefore unstable long-T_1_ tubes are away from the corners and towards the middle of the 3 × 3 array.

### Structural integrity

Inspection of localizers and high-resolution images acquired at baseline and at 12-months post manufacture, revealed no visible air bubbles, gel rips or tears down any of the tubes and images were free of susceptibility artefacts (Fig. 1ii and Additional file 2: Movie S1 and Additional file 3: Movie S2). This inspection was done visually by C.C.T. and G.C. T_1_ and T_2_ maps collected through the midline of the phantom, using the specified scan setup, were free from off-resonance artifacts.

### Characterization of T_1_|T_2_ dependence on agarose and nickel

**Fig. 2:**
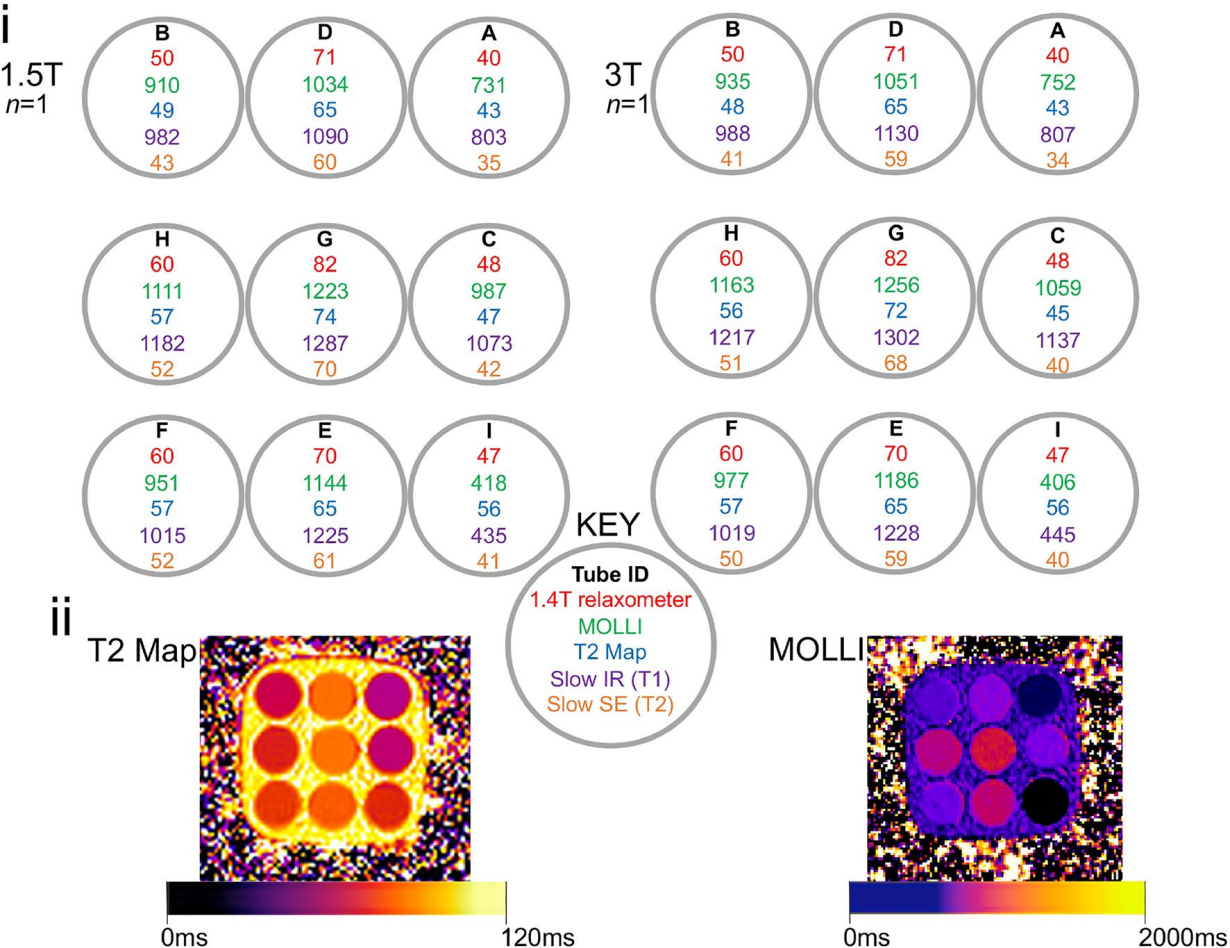
**i** T_1_ and T_2_ (in ms) in the T_2_ phantom (*n* = 1) as measured at 1.5 T and 3 T: slow scan reference T1 obtained using inversion recovery (IR) gradient echo (GRE) (purple) and reference T_2_ using single echo (SE) (orange); T1 via modified Look-Locker inversion recovery (MOLLI) T_1_ mapping (green) and T_2_ via balanced steady state free precession (bSSFP) T_2_ mapping (blue); T_2_ obtained by the manufacturer in Australia using a 1.4 T Bruker minispec relaxometer at 22 °C (red). Tube arrangement is such that the more temperature-dependent and therefore unstable long-T_1_ tubes are away from the corners and towards the middle of the 3 × 3 array. **ii** Exemplar T_2_ and T_1_ maps on a Siemens 3 T Prisma clinical CMR scanner. *ID* = *tube identity*

In the phantom, the T_1_|T_2_ measured on a 1.4 T Bruker relaxometer at 22 °C and on 1.5 T and 3 T clinical CMR systems using IRGRE, SE, MOLLI and T_2_ mapping bSSFP are presented in Fig. 2i. Example T_1_ and T_2_ maps of the phantom are displayed in Fig. 2ii. The 9 compartments successfully covered the clinically meaningful range of native and post-GBCA myocardial T_2_ in health and disease (typically expected to be between 43 ms to 74 ms by T_2_ mapping).

### ***B***_0_ uniformity

**Fig. 3:**
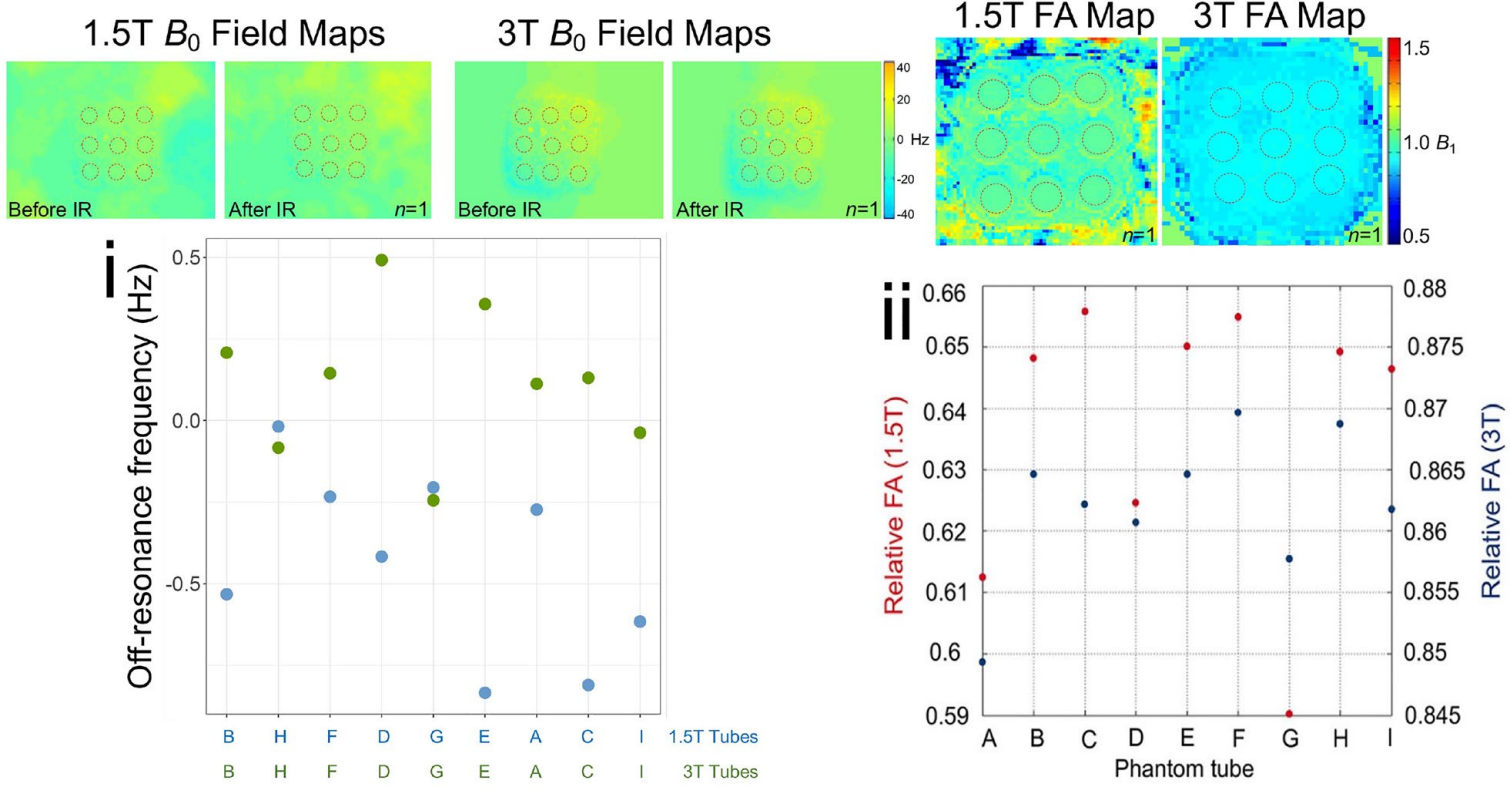
**i** *B*_0_ field homogeneity across the nine phantom compartments as a measure of off-resonance in Hertz (Hz) at 1.5 T (blue) and 3 T (green) are shown (bottom). The associated *B*_0_ field maps with the field of view capturing the whole phantom at 1.5 T and 3 T are also presented (top–tube positions are overlaid in red). ii) *B*_1_ field homogeneity across the nine phantom compartments as a measure of the FA (in degrees) at 1.5 T (red) and 3 T (blue) are shown (bottom). These represent small shifts in FA or frequency (e.g.,10 Hz = 0.08 ppm at 3 T) and should not be regarded as significantly different between the tube compartments. As expected, the variation of relative FA is larger at 3 T (0.590–0.656) compared to 1.5 T (0.849–0.866). The associated *B*_1_ field maps of at 1.5 T and 3 T are also presented (top–tube positions are overlaid in red). *FA* flip angle. Other abbreviations as in Fig. 2

When coaxially aligned with *B*_0_, scanning the phantom at its isocenter halfway along its length (i.e., scan slice-labelled on the phantom exterior) provided sufficient *B*_0_ uniformity. The final phantom was free of off-resonance artifacts when scanned at the isocenter as per the user manual (i.e., bottle placed co-axial with the z axis, use of shimming etc.). Across the 9 tubes, off resonance at both 1.5 T and 3 T was less than 1 Hertz (Hz) (i.e., 0.008 parts per million [ppm] at 3 T or 0.004 ppm at 1.5 T) indicating minimal *B*_0_ distortion. Given these are extremely small shifts, off resonance should not be considered as different between the 9 tubes. The associated 1.5 T and 3 T *B*_0_ field maps are shown in Fig. 3i.

### ***B***_1_ uniformity

Across the nine phantom compartments embedded in the outer matrix fill packed with HDPE beads, there was minimal *B*_1_ field inhomogeneity as a measure of the FA (i.e., less than 0.9; exemplar 1.5 T and 3 T *B*_1_ field maps in Fig. 3ii.

### Reference T_1_|T_2_

**Fig. 4:**
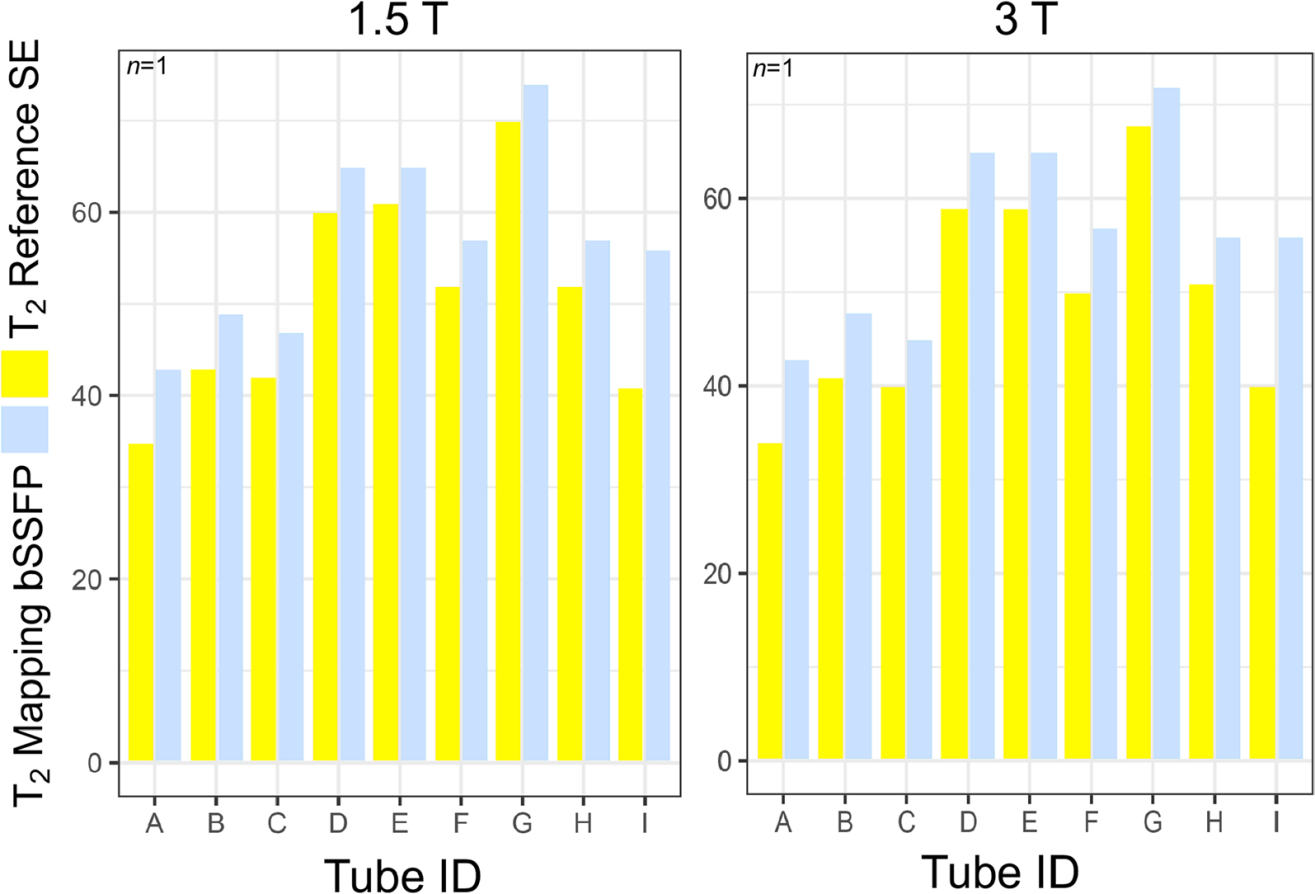
Comparison of T_2_ obtained by reference (long-TR) SE sequences (yellow) versus bSSFP T_2_ mapping (grey) at 1.5 T (Siemens Aera, left) and 3 T (Siemens Prisma, right) on the final phantom (*n* = 1) at baseline. *TR* repetition time. Other abbreviations as in Fig. 2

Baseline reference T_1_ obtained via IR GRE were compared to those obtained by pre-GBCA MOLLI T_1_ mapping (Fig. 2i), while reference T_2_ obtained via SE were compared with T_2_ mapping bSSFP at both 1.5 T and 3 T for each of the 9 tubes Table 2 and Fig. 4.

**Table 2:** Comparison of T_2_ obtained by reference (long-TR) spin-echo sequences versus balanced steady state free precession (bSSFP) T_2_ mapping at 1.5 T (Siemens Aera) and 3 T (Siemens Prisma) on the final phantom (*n* = 1) at baseline

Biological scope (Tube ID)	1.5 T			3 T		
	T_2_ mapping (ms)	Spin-Echo (ms)	Difference in ms (%)	T_2_ mapping (ms)	Spin-Echo (ms)	Difference in ms (%)
Short T_2_ native myocardium at both 1.5 T and 3 T (A)	43	35	**8 (23%)**	43	34	**9 (27%)**
Medium T_2_ native myocardium at 1.5 T (B)	49	43	**6 (14%)**	48	41	**7 (17%)**
Medium T_2_ native myocardium at 3 T (C)	47	42	5 (12%)	45	40	5 (13%)
Long T_2_ native myocardium at 1.5 T (D)	65	60	5 (8%)	65	59	6 (10%)
Long T_2_ native myocardium at 3 T (E)	65	61	4 (7%)	65	59	6 (10%)
Long-normal T_2_ native myocardium at 1.5 T (F)	57	52	**5 (10%)**	57	50	**7 (14%)**
Very long T_2_ native myocardium at 3 T (G)	74	70	4 (6%)	72	68	4 (6%)
Mildly long T_1_ & T_2_ native myocardium at 1.5 & 3 T (H)	57	52	5 (10%)	56	51	5 (10%)
Medium T_1_ & T_2_ post-GBCA* myocardium at 1.5 & 3 T (I)	56	41	**15 (37%)**	56	40	**16 (40%)**

Corner tubes are highlighted in bold

*TR* repetition time. Other abbreviations as in Table 1

Overall, there was a relatively good agreement between T_2_ measured by T_2_ mapping bSSFP and SE with a deviation of 6.3 ms [12.5%] at 1.5 T and 7.2 ms [14.7%] at 3 T. Corner tubes (i.e., A, B, F, and I) displayed a higher deviance at both 1.5 T (8.5 ms [19.9%] vs 4.9 ms [8.7%]) and 3 T (9.8 ms [23.6%] vs 5.5 ms [10.2%]). The difference is mostly driven by the post-GBCA ultra-low T_1_ Tube I (that has a deviance of 15 ms [36.6%] and 16 ms [40.0%] for 1.5 T and 3 T respectively).

### Temperature dependency

**Fig. 5:**
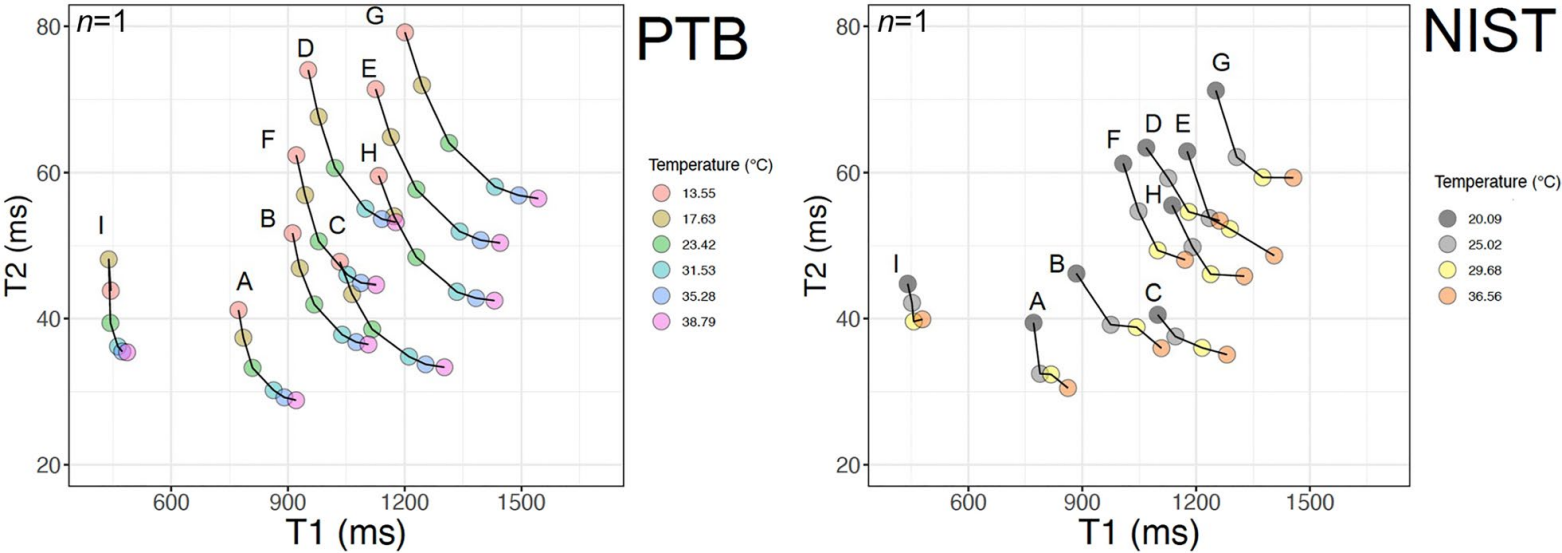
Temperature tests carried out at PTB–German Physikalisch-Technische Bundesanstalt (left)–using a 3 T Siemens Magnetom Verio (VB17) and a 12-channel head coil and at NIST–US National Institute of Standards and Technology (right)–using an Agilent 3 T small bore scanner. T_1_ was measured by IRSE, and T_2_ by SE. The measurements were performed on the final phantom (*n* = 1) at baseline. *TE* echo time. Other abbreviations as in Fig. 2

Temperature tests carried out at PTB and NIST in 3 T scanners, with T_1_ was measured by IR SE, and T_2_ by SE. As the temperature increases, T_1_ increased and T_2_ decreased across the 9 tubes (Fig. 5). Short and medium T_1_|T_2_ tubes were more stable as the variation in the temperature was more pronounced for long T_1_|T_2_ tubes (G, E, H, D).

### Reproducibility

#### Short-term reproducibility

**Fig. 6:**
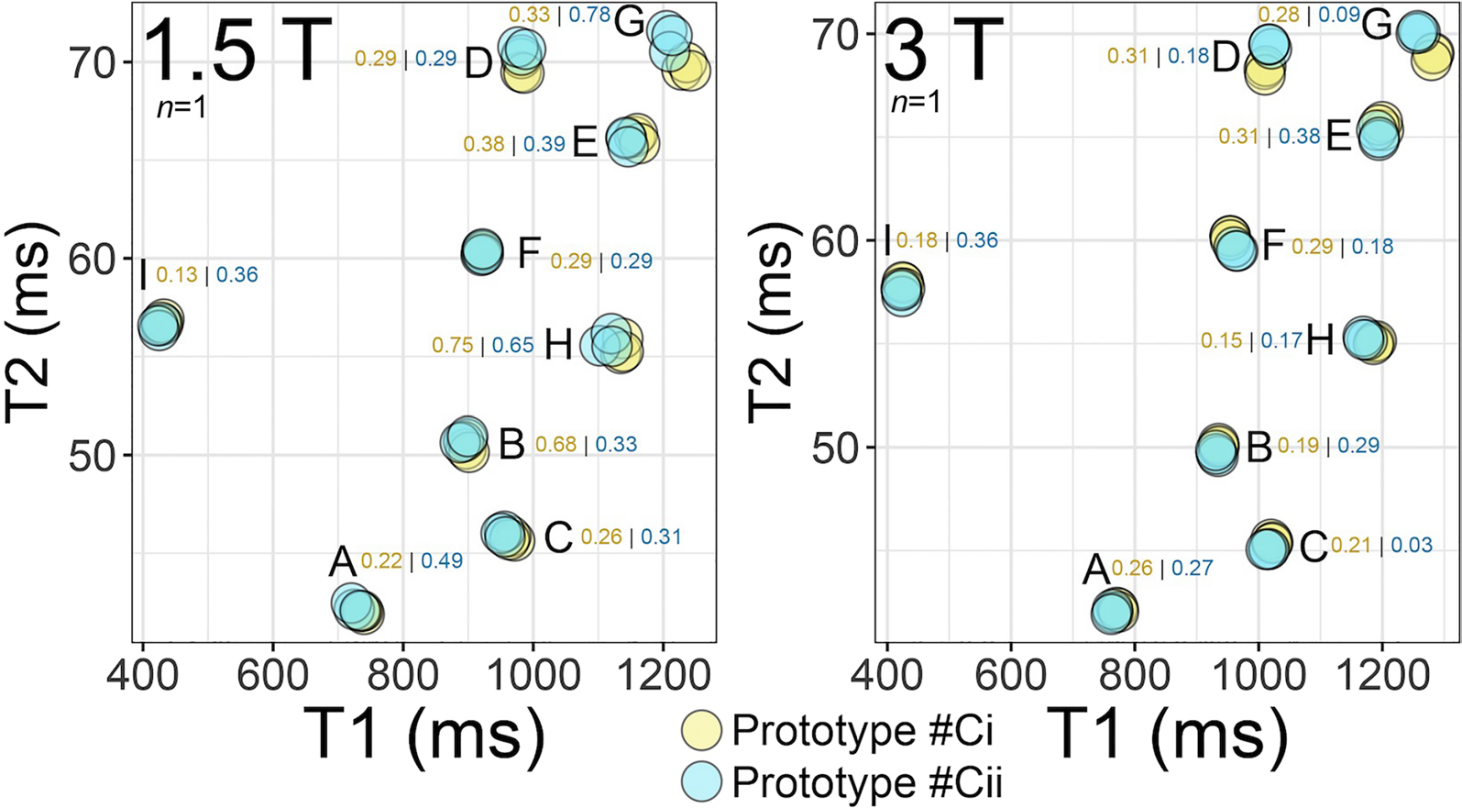
Short-term reproducibility of T_2_ at 1.5 T (left) and 3 T (right) acquired using T_2_ mapping bSSFP repeated 3 times in each of the final prototypes #Ci (at a temperature of 22°) (*n* = 1) and #Cii (at 21°) (*n* = 1) manufactured months apart, from independent stock solutions. All these scans were performed on the same day with independent placement of phantom and shims. Coefficients of variation (CoV) of T_2_ are shown per tube and were all < 1% in the absence of temperature correction. CoV for T_1_ using 3 MOLLI repeats are not shown here but were also < 1% for both prototypes (1.5 T range: 0.13–0.94%; 3 T range: 0.03–0.38%)

All 9 tubes, at both field strengths, showed a CoV of < 1% for both T_1_ and T_2_ reproducibility, even in the absence of temperature correction, and regardless of phantom batch. As expected, tubes D and G with the longest T_1_ and T_2_ showed the greatest variability between repeated scans (Fig. 6).

#### Long-term reproducibility

Between the baseline and 12 month repeat scans, the CoV across all 9 tubes and both field strengths for T_1_ measured by MOLLI T_1_ mapping, was < 1.38% and for T_2_ measured by bSSFP T_2_ mapping was < 1.25% (range of CoV at 12 months for the 9 T_1_ tubes at 1.5 T = 0.01–1.38% and at 3 T = 0.01–1.25%; range of CoV at 12 months for the 9 T_2_ tubes at 1.5 T = 0.00–1.25% and at 3 T = 0.10–1.22%). All measurements were acquired at 22 °C, meaning that no temperature correction was required. There was a greater variability of reads at 3 T compared to 1.5 T, and a greater variability of long T_1_|T_2_ tubes (D and G) compared to other tubes, in line with our previous work [10] (Table 3).

**Table 3:** Long-term reproducibility results for one phantom at baseline and its 12 months repeat scan

	1.5 T *n* = 1		3 T *n* = 1	
Biological scope (Tube ID)	Baseline	12-month repeat (absolute difference in ms, *% diff*)	Baseline	12-month repeat (absolute difference in ms, *% dif*)
Short T_2_ native myocardium at both 1.5 T and 3 T (A)	43	43 (0, *0%*)	43	43 (0, *0%*)
Medium T_2_ native myocardium at 1.5 T (B)	49	49 (0, *0%*)	48	49 (1, *2.1%*)
Medium T_2_ native myocardium at 3 T (C)	47	47 (0, *0%*)	45	45 (0, *0%*)
Long T_2_ native myocardium at 1.5 T (D)	65	66 (1, *1.5%*)	65	65 (0, *0%*)
Long T_2_ native myocardium at 3 T (E)	65	65 (0, *0%*)	65	65 (0, *0%*)
Long-normal T_2_ native myocardium at 1.5 T (F)	57	57 (0, *0%*)	57	57 (0, *0%*)
Very long T_2_ native myocardium at 3 T (G)	74	73 (1, *1.4%*)	72	71 (1, *1.4%*)
Mildly long T_1_ & T_2_ native myocardium at 1.5 & 3 T (H)	57	57 (0, *0%*)	56	56 (0, *0%*)
Medium T_1_ & T_2_ post-GBCA* myocardium at 1.5 & 3 T (I)	56	57 (1, *1.8%*)	56	57 (1, *1.8%*)

All the T_2_ presented in this table were acquired using T_2_ mapping bSSFP in final prototype #Ci at baseline and prototype #Cii at the 12-month repeat

Other abbreviations as in Table 2

## Discussion

In this study, we developed a T_2_ phantom for quality assurance of T_2_ mapping in CMR. By varying the concentrations of agarose and Ni^2+^ we were able to make 9 tubes that covered the relevant spectrum of human myocardial T_2_ (i.e., both native and post-GBCA) across health and disease. At 12 months post manufacture compared to baseline, the phantom remained structurally intact and free of susceptibility artefacts when scanned at the isocenter, with good *B*_0_ and *B*_1_ field homogeneity and small variability in T_1_|T_2_ (all CoV < 1.38%).

T_2_ mapping has gained a lot of traction lately as it enables both the visual identification and quantification of regional and diffuse myocardial disease in a color-coded fashion [2]. T_2_ mapping can be achieved using bright blood sequences such as turbo spin echo [21, 22], multi-echo spin echo [6], gradient spin echo [23], or T_2_-prepared bSSFP [24]. The latter is the most widely used given its accuracy [25] and reproducibility [26]. To overcome the inherent bias to T_1_ of bSSFP, T_2_ preparation (e.g., using the Carr-Purcell Malcom-Levit sequence [27]) can be employed to promote T_2_ weighting (and hence the term T_2_-prepared bSSFP) [28]. Moreover, T_2_-preparred sequences display a reduced field-strength variability [29]. Indeed, in our study the CoV were slightly higher at 3 T compared to 1.5 T.

To date, a gold standard calibration instrument for T_2_ mapping is yet to be established. The design challenges which need to be considered when creating a phantom object for parametric mapping have been previously described [9]. Briefly, these include: (1) recipient shape magnetostatics and *B*_0_ distortion, (2) long term stability, (3) structural considerations (e.g., seal, leakages, and air trapping), (4) adjustments of *B*_0_ and reference frequency, (5) phantom diamagnetism, (6) in plane effects such as Gibbs artifact ringing, (5) field strength performance, (6) biological scope of selected T_1_|T_2_ (i.e., ideally covering clinically relevant pre/post-GBCA myocardial ± blood values in health and disease), and (7) number of compartments and their arrangement. GBCA can shorten both T_1_ (~ 25%) and T_2_ (~ 5%) [30], and T_2_ mapping is usually done pre-GBCA [2]. However, we provisioned for one post-GBCA tube (tube I) on account of its potential research utility for groups working on pre- and post-GBCA multiparametric mapping and CMR fingerprinting. The current data suggest that the proposed CMR T_2_ phantom has adequately addressed all these needs. This was enabled by the expertise gained from the recently completed T1MES® programme [10]. Compared to our T1MES® phantom, the CMR T_2_ phantom: (1) provides improved coverage of the myocardial native and post-GBCA T_2_ in health and disease, (2) is useable at both field-strengths for more flexible and cost-effective utilization by end users, and (3) has a smaller total volume compared to T1MES® to further reduce artefacts. Although the T_2_  phantom covers *some* clinically relevant T_1_ values, the T1MES® phantom provides a more extensive coverage of T_1_ in health and disease, better field strength specificity and dedicated pre- and post-GBCA blood and myocardial T_1_ tubes. Thus, if T_1_ mapping/Extracellular Volume (ECV) quantification quality assurance is being pursued, we still recommend using the T1MES® phantom for such calibration, instead of the T_2_ phantom.

Our T_2_ phantom is partly composed of diamagnetic (the gel and HDPE beads) and paramagnetic (Ni^2+^) constituents, but since the Ni^2+^ concentrations are extremely small, the prevailing interaction of the device with the magnetic field may be considered to be diamagnetic causing a negligible frequency shift of < 1 Hz (equivalent to < 0.008 ppm). Based on our experience with T1MES®, we expect the T_2_ phantom to have a shelf-life of up to 2 years, but currently only a single final phantom has been tested and only up to 12 months.

Based on published cohort studies, 1 SD of the mean native myocardial T_2_ is generally ~ 3 ms at 1.5 T and 3 T [31–33]. Thus, we arbitrarily pre-defined as repeatable (and suitable for clinical/research use), a phantom object where the estimated variance of its serial T_2_ data did not exceed ½ of the above 1 SD. Assuming a typical native myocardial T_2_ of 45 ms and a variance of ≤ 1.5 ms (i.e., ½ of the mean native myocardial T_2_), this yields an acceptable CoV ≤ 2.7%. We go on to show that the long-term (12-month) reproducibility of the CMR T_2_ phantom was in fact of the order < 1.38% (and short-term reproducibility < 1%) when using CoV.

All tubes regardless of whether they have a central or corner position express similar deviances of bSSFP T_2_ compared to SE T_2_ except for post-GBCA Tube I (which has a deviance of 15 ms [36.6%] at 1.5 T and 16 ms [40.0%] at 3 T). Tube I’s extreme bSSFP vs SE deviances are partly due to its ultra-low T_1_ (406 ms) which differentially impacts T_2_ reads by bSSFP vs SE sequences. For the remaining 8 tubes, measurement errors between SE and bSSFP T_2_ tended to be slighty higher in corner when compared to central tubes, at both 1.5 T (6.3 ms [11.5%] vs 4.9 ms [8.7%]) and 3 T (8 [16.2%] vs 5.5 ms [10.2%]).

Moving forward, we anticipate our CMR T_2_ mapping phantom will be able to support multi-center T_2_ mapping studies by allowing sites to measure and compare the stabilities of their local sequence-software combinations and permit comparisons across centres for the pooling data. By highlighting performance discrepancies between T_2_ mapping prototypes and established commercially available products, developers will be compelled to refine their sequences if appropriate, thus advancing the T_2_ mapping field. In addition, we also expect our phantom to pave the way towards local phantom calibration overriding the need for local reference ranges [34].

A mandatory step before transitioning this device into clinical CMR centers for local quality assurance, is the receipt of regulatory clearance. Our applications for clearance by the US Food and Drug Administration (FDA), Conformitée-Europeen (CE) mark in the EU and Therapeutic Goods Administration (TGA) in Australia are in progress.

### Limitations

A limitation of our study is that the number of phantoms tested (i.e., four) was small. Stability was evaluated in one phantom, and reproducibility and accuracy were evaluated in two at a single center, of which one was at 1.5 T and one was at 3 T.

Phantoms have an unrealistically high SNR, are not magnetically representative of tissues as they fail to embody properties such as magnetization transfer, and they do not capture clinically relevant CMR challenges such as partial volume effects at the blood-myocardial interfaces [9, 10, 35]. These factors were beyond the scope of our study which aimed solely to pilot a quality assurance T_2_ mapping phantom. In addition, good in vitro performance in phantom experiments does not guarantee good performance in patients as it fails to capture real-life clinical scenarios (e.g., patients with arrythmias). The temperature sensitivity of the tubes might be problematic in severely hypo- or hyperthermic patients. All the CMR imaging was performed using a single vendor (Siemens) at UCL. The standard Siemens color scale for the T_2_ map was noted to be insufficiently granular within the physiological range. GBCA can shorten both T_1_ (~ 25%) and T_2_ (~ 5%) [30], but the effects vary based on the specific GBCA used. Studying individual GBCA agents was beyond the scope of this study. Higher concentrations of the paramagnetic Ni^2+^ would have been required to capture physiologically relevant T_2_^*^ at the cost of *B*_0_ distortion. As this would have reduced our ability to model T_2_, quality assurance of T_2_* mapping was not pursued in this work.

The overall purpose of phantoms is to create a reproducible set of T_1_|T_2_ that can be used to calibrate imaging sequences within a site longitudinally and at different sites. In this paper, we defined adequacy in terms of *B*_0_|*B*_1_ uniformity and T_1_|T_2_ precision rather than T_1_|T_2_ accuracy, given the known differences between SE and bSSFP or other vendor-specific T_2_ mapping readouts, particularly in the context of varying tube-T_1_ and temperature. Given myocardial T_2_ ranges from about 43 to 74 ms in health, the average absolute deviations between SE and T_2_ bSSFP (i.e., 6.3 ms at 1.5 T and 7.2 ms at 3 T) represent ~ 20% of the physiological range except for the ultra-low T_1_ tube I whose deviation represents ~ 50%. When evaluating accuracy, focusing solely on the absolute difference in ms between T_2_ measured by T_2_ mapping bSSFP and SE can be misleading. However, percentage differences are also provided. A cylindrical phantom with greater edges-tubes spacing may have led to a better T_1_|T_2_ accuracy but this would have been associated with more off-resonance artefacts. Although the 9 stock solutions for tubes are reproducibly specified, slight inter-batch differences are to be expected as with all nickel-chloride/agarose solutions, even when formulated using rigorous protocols. This is why each new stock solution, undergoes de novo 1.4 T Bruker relaxometer at source, meaning these T_1_ and T_2_ values can be shared with the receiving centres to serve as a benchmark for cross-site comparisons between batches. Lastly, inter-center reproducibility was not addressed, and the reported short and long-term reproducibility data were based on single-centre results from one final T_2_ phantom serially examined at UCL. Since it is of vital importance in the phantom’s transition to clinical practice, this will be the focus of our future work.

## Conclusion

We have reported on the development and testing of a T_2_ mapping phantom demonstrating good structural integrity, *B*_0_/*B*_1_ uniformity, reproducibility and coverage of the clinically relevant myocardial T_1_|T_2_ across health and disease. This device may now be mass-produced to support the quality assurance of T_2_ mapping in clinical and research practice.

## Supplementary Information

**Additional file 1.** T2 phantom for quality assurance of T2 mapping user manual.

**Additional file 2: Movie S1.** High-resolution imaging of the T2 phantom at baseline and at one-year post-manufacture at 1.5 T revealed no visible air bubbles, gel rips or tears down any of the tubes and images were free of susceptibility artefacts.

**Additional file 3: Movie S2.** High-resolution imaging of the T2 phantom at baseline and at one-year post-manufacture at 3 T revealed no visible air bubbles, gel rips or tears down any of the tubes and images were free of susceptibility artefacts.

## Data Availability

The supplementary material provided contains detailed information about the T_2_ mapping phantom set-up, use and analysis. Sequence protocols are available on GitHub (https://github.com/gcaptur/T2-Mapping-for-CMR).

## Abbreviations

bSSFP: Balanced steady state free precession
CMR: Cardiovascular magnetic resonance
ECG: Electrocardiogram
FA: Flip angle
GBCA: Gadolinium based contrast agent
GRE: Gradient echo
HDPE: High-density polyethylene
IR: Inversion recovery
MOLLI: Modified Look-Locker inversion recovery
NIST: National Institute of Standards and Technology Laboratory
PC: Polycarbonate
PTB: Physikalisch-Technische Bundesanstalt
PVC: Polyvinyl chloride
RF: Radiofrequency
SCMR: Society for Cardiovascular Magnetic Resonance
SE: Spin echo
T1MES: T1 Mapping and Extracellular Volume standardization
TE: Echo time
