# Supplementary material for: Developing a medical device-grade T2 phantom optimized for myocardial T2 mapping by cardiovascular magnetic resonance
Source: J Cardiovasc Magn Reson. 2023 Mar 20;25:19. doi: 10.1186/s12968-023-00926-z (PMC10026458; doi:10.1186/s12968-023-00926-z)
Supplement: Supplementary file 1 — Additional file 1. T2 phantom for quality assurance of T2 mapping user manual. [file 12968_2023_926_MOESM1_ESM.doc]

T2 PHANTOM for quality assurance of t2 MAPPING USER MANUAL

TABLE OF CONTENTS

ABBREVIATIONS LIST [2](#__RefHeading___Toc303826148)

INTRODUCTION [3](#__RefHeading___Toc303826149)

1 | PHANTOM OVERVIEW [4](#__RefHeading___Toc303826150)

2 | PHANTOM CARE [5](#__RefHeading___Toc303826151)

3 | INSTRUCTIONS FOR PHANTOM SETUP [5](#__RefHeading___Toc303826152)

3.1 | TEMPERATURE [5](#__RefHeading___Toc303826153)

3.2 | ORIENTATION [6](#__RefHeading___Toc303826154)

4 | PHANTOM SCANNING [7](#__RefHeading___Toc303826155)

4.1 | SCAN SETUP [7](#__RefHeading___Toc303826156)

5 | SHIM SETTINGS AND SHIM VOLUME [9](#__RefHeading___Toc303826157)

6 | RESULTS [11](#__RefHeading___Toc303826161)

7 | APPENDIX [12](#__RefHeading___Toc303826162)

A7.1 | **SIEMENS**-specific instructions [12](#__RefHeading___Toc303826163)

A7.2 | **PHILIPS**-specific instructions [14](#__RefHeading___Toc303826164)

A7.3 | **GE**-specific instructions [16](#__RefHeading___Toc303826165)

ABBREVIATIONS LIST

AP = antero-posterior

CMR = cardiovascular magnetic resonance

ECG = electrocardiogram

FOV = field of view

GE = General Electric

HF = head-foot

HDPE = high-density polypropylene

ID = phantom identity number

LCD = liquid crystal display

NiCl_2_ = nickel chloride

PC=polycarbonate

PMU = physiologic monitoring unit

PVC = polyvinyl chloride

P(P)NS = predicted peripheral nerve stimulation

RL = right-left

SAR = specific absorption rate

T = tesla

**INTRODUCTION**

Above all, the aim is consistency each time the phantom is setup and measured. These instructions mainly attempt to keep everything the same every time: phantom orientation and position, coil setup, heart rate, factors affecting scanner calibrations such as the “shim” volume, shim methods, etc. Please try to keep this basic aim in mind. Although running the *B_0_* “shim” is arguably undesirable please note this is specified and should be operated in the same mode with the same receiver coils for it, and the same calculation or fitting volume used each time.

The arrangement of tubes in the phantom is not random: it avoids placing long-T1|T2 tubes in the corner positions where *B_0_* and *B_1_* distortions associated with the phantom are greatest. Alignment of tubes with the *B_0_* direction and scanning halfway along them, not towards their ends, are also important factors in avoiding measurements in regions using distorted fields.

Please do not add “loading” phantoms.

This manual suggests using an R-R interval of 900ms (heart rate 67bpm). Depending on your T2-mapping sequence, an R-R of 800ms or 1000ms may still run repeatably reliably. Provided that the SAME R-R value is used for each session this would be acceptable if 900ms cannot easily be obtained.

1 | PHANTOM OVERVIEW

1. The phantom consists of an amber plastic bottle sealed by a tight black cap.

**🛆 NEVER OPEN THE CAP**

1. Inside the bottle is an agar/nickel chloride (NiCl_2_)/high-density plastic bead fill containing a 3 X 3 array of plastic tubes filled with the T1/T2 mixtures. The tubes rest on a resin layer at the base (**Figure 1**).
2. There is a label with a red isocenter cross on the front surface of the bottle to aid positioning in the scanner.
3. There is another label on the back of the bottle that contains the unique phantom identity number (ID).
4. Please use the Phantom ID for all data entry.
5. Along the top of the bottle’s front surface, is a liquid crystal display (LCD) thermometer strip.

**Figure 1.** External and internal features of the T2 phantom.


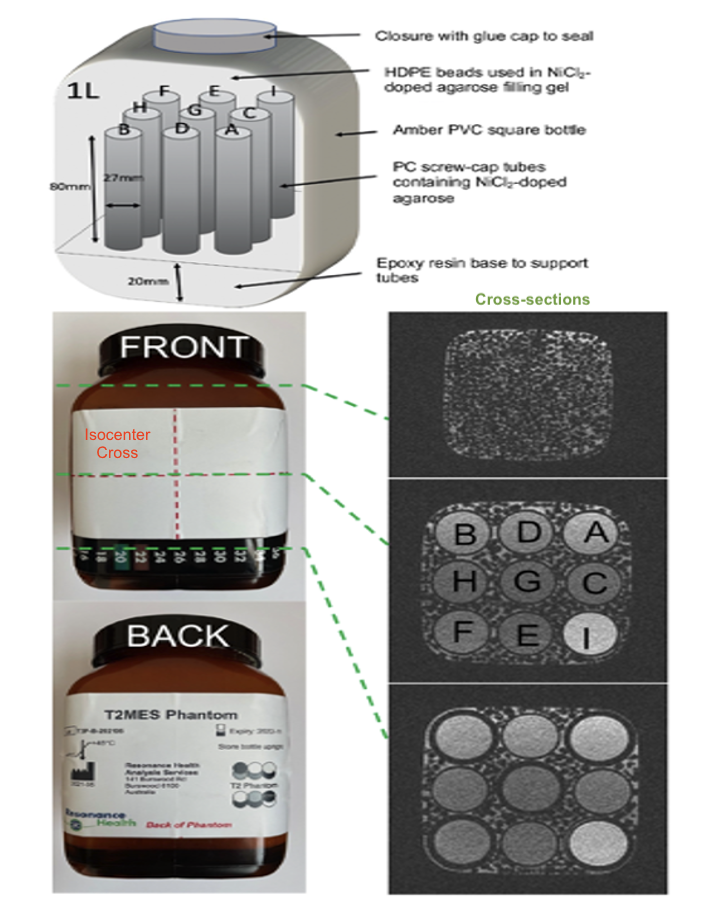


2 | PHANTOM CARE

1. On receiving the phantom, unpack and inspect it. If damaged, contact the manufacturer.
2. Please be gentle when moving the phantom around - do not drop or shake it.
3. Do not place heavy objects on top of or strongly against the phantom at any time (the phantom outer bottle is quite flexible).
4. In between research scans, store the phantom upright in your scanner room for temperature consistency with scanning, and in the same protected area each time.

**🛆 STORE THE PHANTOM VERTICALLY UPRIGHT**

3 | INSTRUCTIONS FOR PHANTOM SETUP

3.1 | TEMPERATURE

1. There is a self-adhesive LCD temperature strip with temperature range 10°C to 40°C along the upper surface of the phantom (**Figure 2**). Temperature markers appear at an interval of 2°C, but the **resolution of the strip is actually 1°C**.
2. Take note of phantom temperature **before** starting each new scan.
3. If there is a bright **GREEN** cell, this marks the exact phantom temperature (it will be an even number).
4. If there is no green cell but adjacent **BLUE** and **TAN** cells, then exact phantom temperature is the odd number **in between** (**Figure 3**).
5. Phantom temperature must be added to the <Patient Name> when **registering the new scan** on your MRI scanner (see 4.1).

| **Figure 2.** Temperature strip | **Figure 3.** If **GREEN** cell = take this value.  If adjacent **TAN/BLUE** cells = take middle value. |
| --- | --- |
| 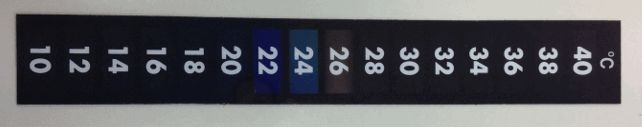 | 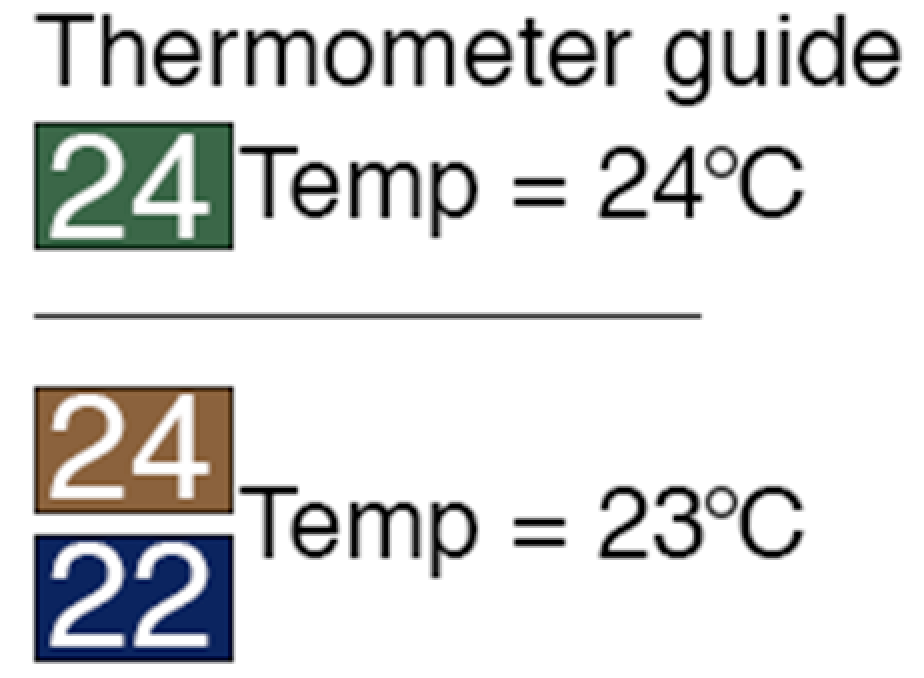 |

3.2 | ORIENTATION

**🛆AIM TO POSITION THE PHANTOM AT ISOCENTER USING THE SAME SUPPORTING MATERIALS IN EACH SCANNING SESSION**

(+/-1cm across [*x*] and along [*z*] the bed; +/-3cm for height [*y*])

1. Set up and scan the phantom at the isocenter position and in **exactly the same** way each time for repeat scans.
2. Read your manufacturer-specific instruction in the **Appendix** for where along the table to place the phantom, i.e., in the *z* direction (for Siemens see A6.1.1; for Philips see A6.2.1; for GE see A6.3.1).
3. Do not place the phantom directly onto the spine or bed coil. To bring it up to isocenter you must elevate the phantom bottle above the spine or bed coil by using a firm support.
4. This height depends on your scanner model and MRI table options. (For Siemens see A6.1.1; for Philips see A6.2.1; for GE see A6.3.1). Test the height of the firm support to find one that will lift your phantom to isocenter (see **Figure 4**).

**🛆USE THIS SAME FIRM SUPPORT FOR ALL SUBSEQUENT SCANS.**

1. The firm support must be **FIRM**. It cannot be so soft that it will deform erratically and prevent consistent phantom positioning (i.e., avoid ordinary pillows and blankets for this application).
2. Suggested materials to use as firm support include:

a. A stack of glued (not stapled) spine journals.

b. A firm supporting (foam plastic) slab that may come with your scanner equipment.

1. Once you configure the correct firm support STORE it in the scanner room with the phantom.
2. Lay the T2 phantom onto the firm support as in **Figure 4**.

**🛆 STORE AND ALWAYS USE THE SAME FIRM SUPPORT FOR EACH SCAN**

**Figure 4.** Phantom (orange) setup on the table (the red line is at isocenter).

**
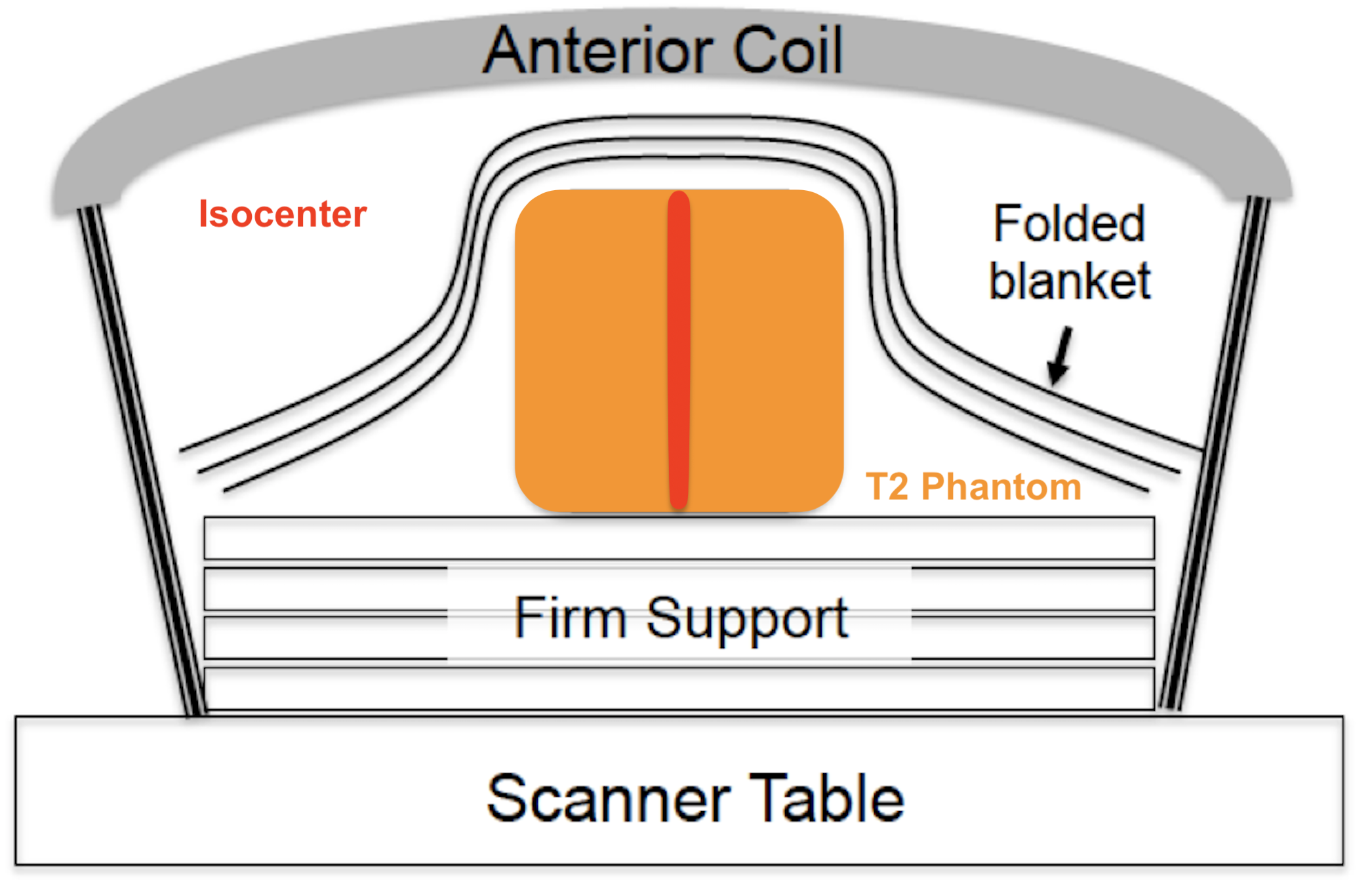
**

1. Consistent elevation will optimise the height of T2 phantom’s isocenter so that you obtain repeated T2 phantom data with CONSISTENT tube position and orientation over time.
2. Place the firm support in the middle of the table’s receiver coil relative to *x*-axis and between the correct table markers for where you would usually position the patient’s heart.
3. The **black bottle cap** (equivalent to the ‘**Head**’) for the specified head-first registration of patient orientation must point into the bore. The isocenter cross sticker must be on the ***upper surface*** of the bottle.
4. ***Before doing anything else***, move the table and shine the laser target markers onto the phantom arrangement till the laser markers are aligned with the red isocenter cross. The pair of *x*-lines and the pair of *z*-lines should overlap.
5. **Memorize phantom temperature now and before introducing bottle into the bore**.
6. Obtain a thick blanket, folded over into a generous rectangular shape and drape this over the top of the phantom. Do this ***after*** having isocentered with lights, ***but before*** applying the surface coil.
7. If possible, use the same anterior cardiac coil for all subsequent phantom experiments.
8. During the subsequent steps, note that the phantom may accidentally be shifted or twisted, and this **should be avoided**.
9. Place the anterior coil onto the blanket that is overlying the phantom with laser target marker still turned on. Adjust the position of the anterior coil manually (do not move the table nor inadvertently slide the phantom) till the ***isocenter cross of the coil*** is aligned with the laser target markers.
10. Strap the anterior coil into place to prevent it slipping off the phantom arrangement. Tighten the straps to secure the blanket around the T2 phantom bottle.
11. While unlikely to matter, please use consistent air-flow settings for repeated scans (e.g., **set the patient fan in the scanner to medium** each session).
12. Once you configure the correct firm support, STORE it in the scanner room ready for repeated use with the phantom.

4 | PHANTOM SCANNING

4.1 | SCAN SETUP

1. Set up the simulated electrocardiographic (ECG) signal. This user manual will prescribe an RR interval of 900ms, but if necessary, other R-R intervals could be used. For Siemens see A6.1.2; for Philips see A6.2.2; for GE see A6.3.2.
2. Before starting the scans make sure **all relevant coils are** **switched on.**
3. Start with scout or localizer imaging. For simplicity it is best if these localisers are all **acquired at isocenter with no image-plane or field of view (FOV) offsets away from isocenter**. Note that: **preset localiser protocols often contain such FOV-shifts or offsets** as typically suiting average patient morphology in the registered patient orientation. It may therefore be easier if you **TURN OFF/UNCHECK** any such FOV-shifts/offsets before running the scout.
4. Check that the physical positioning of the T2 phantom is correct (along all three directions) as follows and that the phantom is not tilted or twisted (for system specific instructions on this see Appendix).
5. Isocentering the T2 phantom along the head-foot (HF) direction (red lines in **Panels A** and **B** in **Figure 5**) should simply be governed by the laser marker. Similarly, isocentering the bottle along the supine patient right-left (RL) direction (yellow lines in **Panels B** and **C** in **Figure 5**) should again simply be governed by the laser marker.
6. Using the firm support, the T2 phantom should be at the correct **height** such that the isocenter is halfway up the central tube, and at the level of the blue line in **Panels A** and **C**, **Figure 5**.
7. In **Panel C** (**Figure 5**) along the yellow line (i.e., height [*y*]), if the isocenter is further than **3cm** from the center of the middle tube, then **positioning is not acceptable**. Please pull out the table, ***change the height of the firm support*** and try again. Once you obtain the correct support for your scanner/phantom arrangement remember this and if possible, **store the materials used with the phantom**, so that repeated scanning is less painful!
8. In the other two directions (along the patient bed [*z*] and across the patient bed [*x*]) the center of the middle tube should be **<1cm** from isocenter. This should be easy to achieve using the laser guidelines and isocenter cross.
9. Please check that the phantom is not tilted or rotated in any axis beyond 10 degrees.

**Figure 5.** Achieving perfect isocenter position of T2 phantom.

*Note that this does not depict any particular scanning software and is for guidance in interpreting the isocenter graphics in use on your particular machine.*


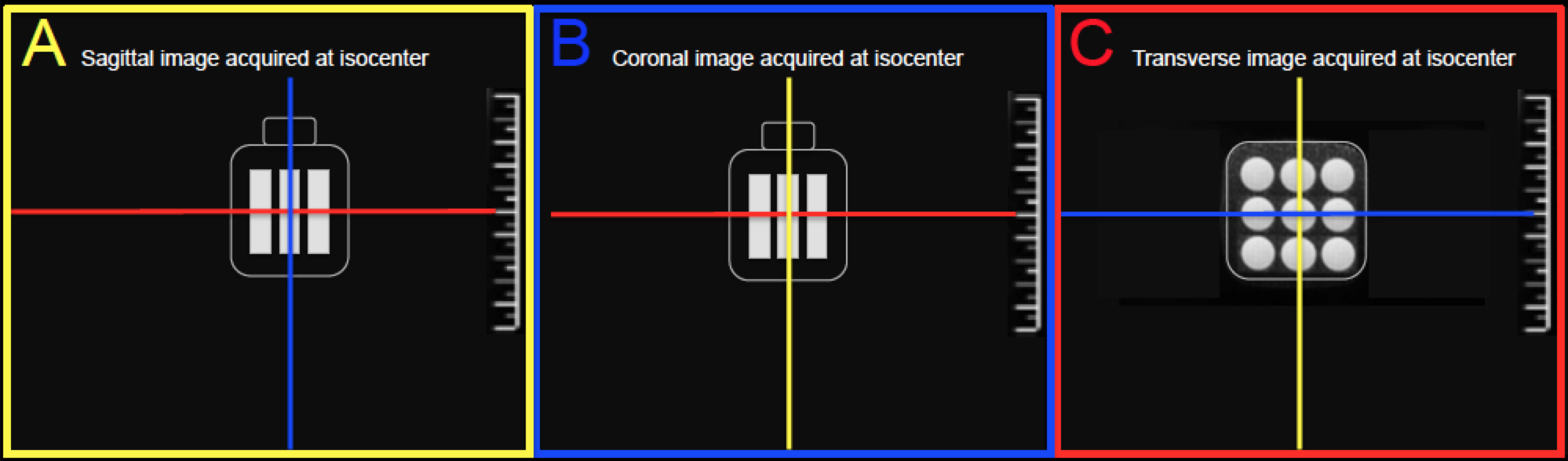


5 | SHIM SETTINGS AND SHIM VOLUME[[1]](#footnote-2)

**🛆 BE CAREFUL TO SET THE SHIM VOLUME AS SPECIFIED IN THIS MANUAL**

1. Before running any T2 mapping sequence you must select the correct shimming method and specify a particular shim volume over the T2 phantom. Please follow the manufacturer-specific instructions for these steps (For Siemens see A6.1.3; for Philips see A6.2.4; for GE see A6.3.4).
2. Apply the same shim volume before starting each set of experiments in a reproducible manner.
3. The shim volume should be positioned at isocenter.
4. **Remember to wait at least 10 seconds with no scanner activity whatsoever before running the first T2 map on your phantom bottle**.
5. Now run your T2 mapping sequence positioned transversely and positioned with FOV centered exactly at isocenter in all three directions.
6. **Remember to wait at least 10 seconds with no scanner activity whatsoever before running any further T2 map on your phantom bottle**.

6 | APPENDIX

A6.1 | **SIEMENS**-specific instructions

A6.1.1 | Table positioning of phantom for SIEMENS

1. For Siemens the bottle should be positioned between **S1 and S2 marker levels** (or if using the 32-channel posterior array consistently center on that array). This is not essential, but we ask as this is easily replicated on each session in case of any unforeseen sources of variability (e.g., there is some metal in the patient table which is known to affect some sequences).
2. Our tests on a 3T Siemens Prisma with a detachable couch have suggested that a firm **support of height 10.5cm** provides the right height to achieve T2 phantom isocenter positioning along the *y* axis.
3. The height for your particular Siemens machine, table and couch settings ***may vary*** so please test firm support height to find your ideal setup (then remember it and if possible store it with phantom!).
4. If your firm support height is correct and isocentering by laser target markers was done carefully, you should expect to see transverse (axial) images similar to those in **Figure A1** when you open your T2 mapping sequence after running the scouts.
5. Aim for the middle of the T2 phantom to be within +/-1cm of isocenter across the bed and within +/-3cm for height (but consistent to within +/-1cm each repeated session).

**Figure A1.** Confirmation of good isocentering of the T2 phantom in Siemens (same for Philips).


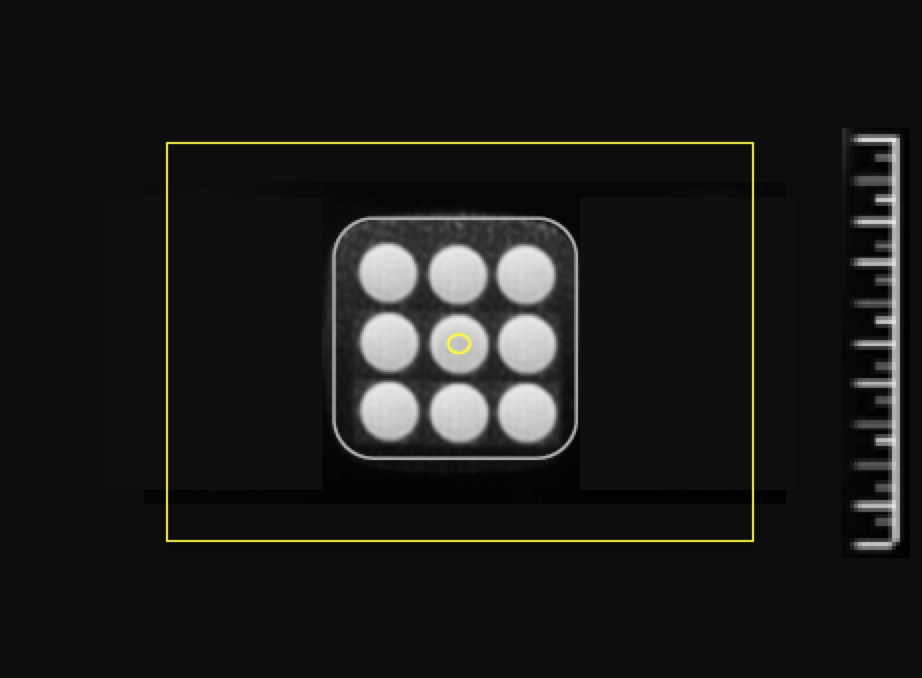


A6.1.2 | Setting up simulated ECG for SIEMENS

1. You will need the Advanced User mode (password is typically <meduser1> for Siemens).
2. Start with <Ctrl-Esc> to launch the <Windows Start> menu.
3. Chose <Run> option.
4. Type: ideacmdtool
5. <Enter>
6. Type: 1
7. <Enter> to Start PMU (physiologic monitoring unit) control.
8. Type: 1 (or ‘4’ on >VD-level software)
9. <Enter> to start ECG simulation.
10. For <ECG period in ms [1000]:>
11. Type: **900**
12. For <RESP period in ms [3000]:>
13. <Enter>
14. For <PULSE period in ms [1000]:>
15. Type: **900**
16. For <EXT period in ms [2000]:>
17. <Enter>
18. Check that the ECG waveform parameters generated are HR=**67**bpm and RR period =900ms.
19. You may now close the black “ideacmdtool” window (you do not need to remember to turn off the simulated ECG settings by using <ideacmdtool> again after the end of your phantom scans to allow the resumption of normal clinical scans, as a new <Patient Registration> will automatically clear it).

**🛆 FOR SIMULATED ECG PLEASE ALLOW TIME FOR SOFTWARE-“AVERAGED” HEART RATE** (if your system uses it) **TO SETTLE, BEFORE STARTING T2 MAPPING**

A6.1.3 | Shim settings for SIEMENS

1. Navigate to <System>
2. <Adjustments>
3. Next to <B0 Shim mode> choose the option <Cardiac>
4. If you do not have the option <Cardiac> available on your Siemens system, then choose <Standard>
5. Make sure that the option <Adjust with body coil> is **ticked**.

A6.1.4 | Adjust volume for SIEMENS

1. Go to <System> and then to <Adjust Volume>.
2. Next to <! Position> select from dropdown list <Isocenter>.
3. Please apply the shim volume tightly around the phantom bottle as shown in **Figure A2**. It should be set 5cm thick in the HF direction and it should follow (+/-2cm) the size of the T2 phantom bottle in the RL and antero-posterior (AP) directions. Adjust the size of the green volume as follows:
   1. Set AP to **100** (mm)
   2. Set RL to **100** (mm)
   3. Set HF **thickness** of the shim slab to **50** (mm).
4. Prescribe the size of the volume in the 3 orthogonal planes to ensure consistent adjustments of *B_0_* and scanner reference frequency over the phantom each time you scan it.
5. Acquire the T2 mapping sequence at isocenter with the T2 phantom right in the middle of the adjust volume.

**Figure A2.** Applying the adjust volume over T2 phantom for Siemens (same for Philips).

***
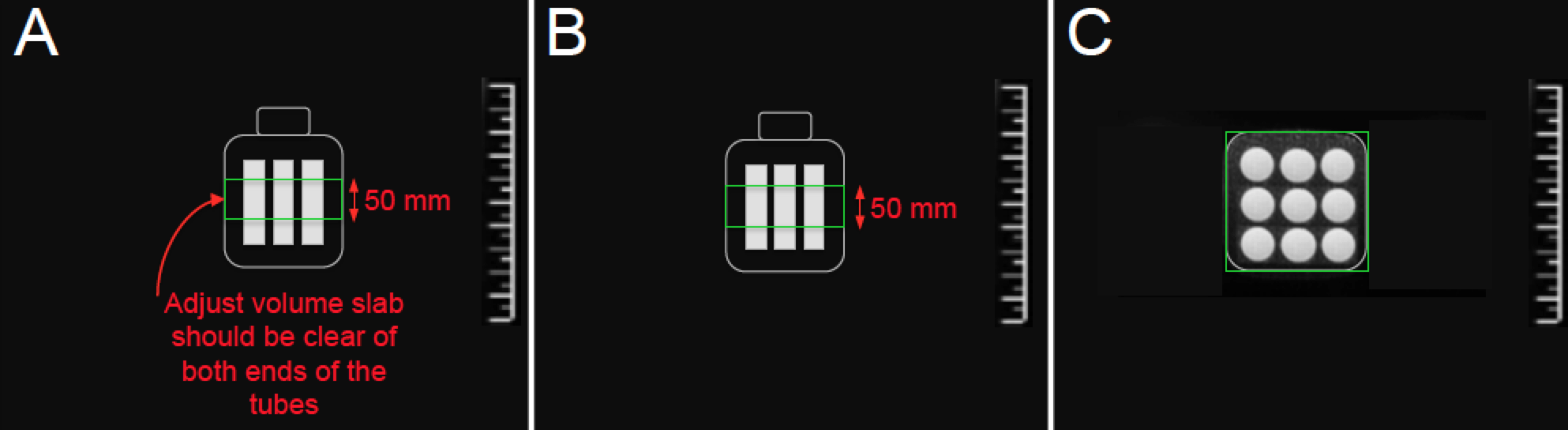
***

A6.2 | **PHILIPS**-specific instructions

A6.2.1 | Table positioning of phantom for PHILIPS

1. The T2 phantom isocenter cross should be placed at the level of the MIDDLE side marker on the Philips table (there are side markers denoting the top, middle and bottom of the elements encased in the coil base).
2. Our previous tests on a Philips Achieva suggested that 10cm was the optimal height to achieve T2 phantom bottle isocenter, ***however this may vary***. To identify the correct height for your particular Philips machine, table and couch configuration please test firm support height to find your ideal setup (then remember it and if possible, store it with phantom!).
3. If your firm support height is correct and isocentering by laser target markers was done carefully, you should expect to see images like those in **Figure A1** (in previous **section A6.1.1**) when you open your T2 mapping sequence after running the scouts.

A6.2.2 | Setting up simulated ECG for PHILIPS

1. Right click the main window and select <Control Parameter Editor>.
2. Select <General Tab>.
3. Select <Physiology Simulation>.
4. Select <Yes>.
5. Set the number of RR intervals = 1
6. Set RR interval (in ms) = **900**
7. Click <Apply>.
8. Remember to turn off the simulated ECG settings after the end of your phantom scans to allow the resumption of normal clinical scans.

**🛆 FOR SIMULATED ECG PLEASE ALLOW TIME FOR SOFTWARE-“AVERAGED” HEART RATE** (if your system uses it) **TO SETTLE, BEFORE STARTING T2MAPPING**

A6.2.3 | Before running a T2 Map that involves SENSE in PHILIPS

1. Before setting up your mapping protocol it is your responsibility to check the control parameters of your Philips platform.
2. Ensure control parameters on Philips are set to DEFAULT VALUES.
3. Ensure no "patches" that could impact scanner behaviour unexpectedly are installed, except of course the patch to run your T2 mapping sequence if you need this.
4. Allow the scanner to perform the SENSE/CLEAR reference scans if using a T2 mapping sequence with parallel imaging.
5. If it asks about **PPNS** and **SAR** allow 1^st^ Level.

A6.2.4 | Shim settings for PHILIPS

1. To set shim mode on Philips select <Contrast> tab.
2. Locate <Shim> in the column of parameters on the left.
3. Select option <Volume>. **CAUTION** - please do not use other “shim” options and if in doubt please ask for advice.
4. Next adjust the green volume slab: Please apply the shim volume tightly around the phantom bottle as shown in **Figure A2** (in previous **section A6.1.5**). It should be set 25mm thick in the HF direction and it should follow (+/-2cm) the size of the phantom bottle in the RL and AP directions.
5. Go to the tab <offc/ang>.
6. Under <Shim Size> adjust the size of the green volume as follows:
   1. Set AP to **100** (mm)
   2. Set RL to **100** (mm)
   3. Set HF **thickness** of the shim slab to **25** (mm).
7. Prescribe the size of the volume in the 3 orthogonal planes to ensure consistent shim and reference frequency estimation over the phantom each time you scan it.
8. Acquire the T2 mapping sequence at isocenter with the T2 phantom right in the middle of the adjust volume.

A6.3 | **GE**-specific instructions

A6.3.1 | Table positioning of phantom for GE

1. Our previous tests on a 3T GE 750 have suggested that a firm support of height **5cm** provides the right height to achieve phantom isocenter positioning along the *y* axis.
2. The height for your particular GE machine, table and couch configuration ***may vary*** so please test firm support height to find your ideal setup (then remember it and if possible, store it with phantom!).
3. If your firm support height is correct and isocentering by laser target markers was done carefully, you should expect to see images similar to those in **Figure A3** when you open your T2 mapping sequence after running the scouts.
4. Aim for the middle of the T2 phantom to be within +/-1cm of isocenter. If it’s perfectly correct you will see an <X> mark in the middle tube on the GE scanning software. A +/-3cm offset on the vertical (*y*) axis is acceptable but whatever height is achieved should be replicated each session to within +/-1cm. Both horizontal directions (across [*x*] and along [*z*] the patient bed) should be easily within +/-1cm using the laser guides and isocenter cross sticker.

**Figure A3.** Confirmation of good isocentering of the T2 phantom in GE.


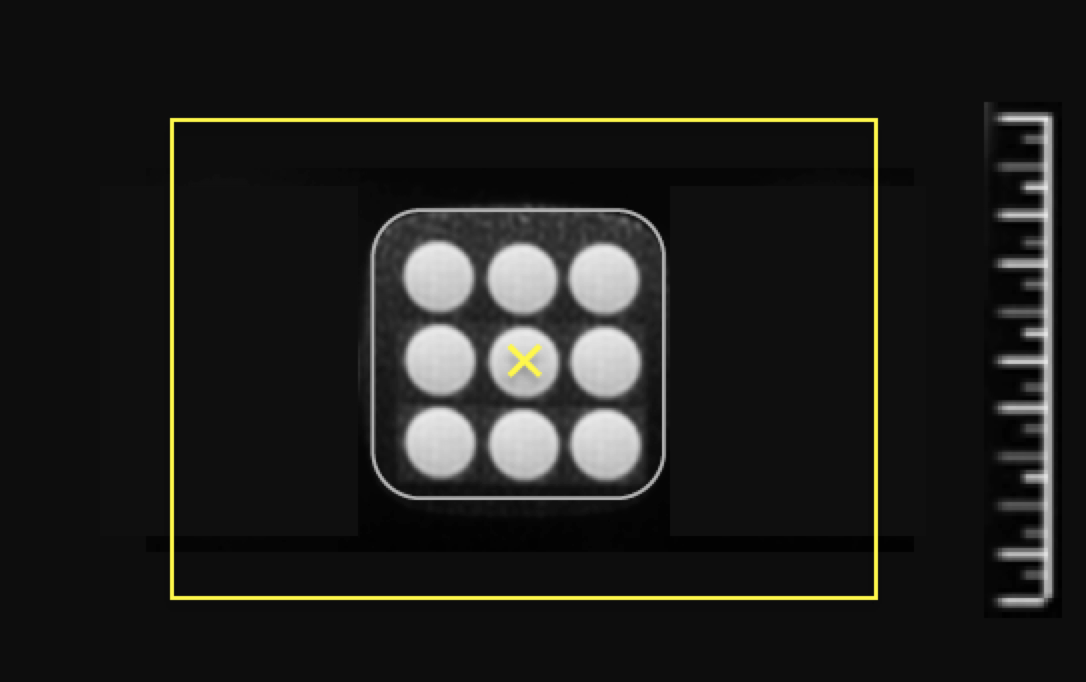


A6.3.2 | Setting up simulated ECG for GE

1. **OUR PREFERENCE** is that you use (or purchase, if you do not already have one) **a basic ‘ECG simulator’ (also known as ‘Chicken Heart’)**. Work with CAUTION this ECG simulator may be dangerous in the magnet room.
2. Set heart rate on the device to **67bpm**.
3. Please position the ECG simulator a safe distance from the bore and follow the manufacturer’s instructions.
4. The **less preferred** alternative is to use the command window to set the ECG Simulator on GE to 80bpm.
5. Go to <Tools> menu (this is the icon at the top left showing a spanner/hammer).
6. Click on the icon’s arrow to reveal the drop down.
7. Select <Command Window>.
8. Type: rlogin scp
9. <Enter>
10. Login: scp
11. Password: scpservice
12. Type: SCP_LX->EmulatePac
13. <Enter>
14. This sets a simulated ECG with rate 100bpm (this heart rate is too fast for the T2 mapping planned. Siemens and Philips users will be using a simulated ECG of 67bpm).
15. To slow down the heart rate (imperfectly) go to back to <Tools> menu and hit the actual button.
16. Select <Open Gating Control Window>.
17. <Waveform display> (select all).
18. By setting <Trigger Lead> to <PG> heart rate will slow from 100bpm to 80bpm – it is still imperfect as the heart rate of 67bpm cannot be achieved; hence why we recommend the ‘Chicken Heart’. Either way, please use the same heart rate for all sessions!
19. If you have set the simulated ECG using the command window in GE, please allow time for any software-“averaged” heart rate (if your system uses it) to settle, before starting T2 mapping work.
20. If you have set the simulated ECG using the command window in GE, remember to disable it when done scanning to permit resumption of routine clinical scans. To disable simulated ECG: hit <Tools> icon.
21. Select <Go to Service Desktop Manager> and hit <TPS Reset> to REMOVE the simulated ECG and reset the gating.

A6.3.3 | Before running a T2 Map that involves SENSE in GE

1. Select to run <**First Level Mode**> if prompted.

A6.3.4 | Shim settings for GE

1. Shim volume in GE is shaped by default as an obligatory cube.
2. To adjust its size go to <Graphic Rx Toolbar> (if you do not immediately see the Toolbar, hit the <GRx> tab).
3. Click <Shim>.
4. Uncheck the <Hide Shim> option to reveal the shim cube (it will look like a green/yellow hashed cube).
5. Next to <FOV> enter <10> to set the size of the cube to 10 X 10 X 10cm.
6. Move the cube so that it covers the middle lengths of the tubes and try to avoid the edges at either end as in **Figure A4**.

**Figure A4.** Applying the adjust volume over the T2 phantom for GE.

***
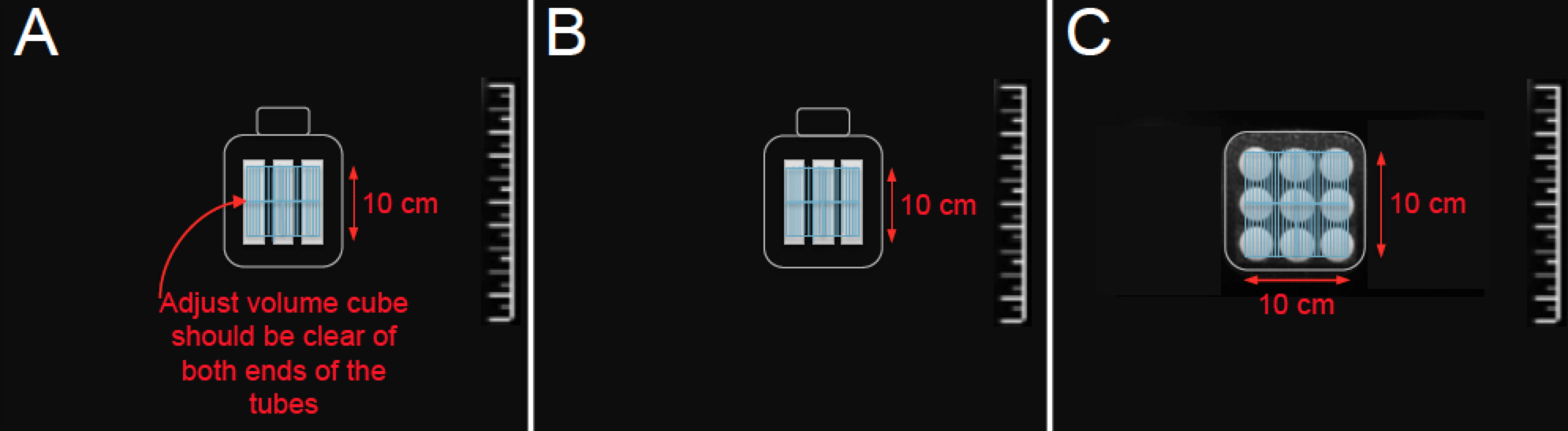
***

***Thank you***

1. *N.B. ‘Shim Volume’ = shorthand for adjustments volume, adjust region, shim region, shim box. See manufacturer-specific Appendix for more details.* [↑](#footnote-ref-2)
